# *CASP1* as a key autophagy-related gene in atherosclerosis: Identification by bioinformatics analysis and experimental validation

**DOI:** 10.1371/journal.pone.0353352

**Published:** 2026-08-19

**Authors:** Han Gao, Yuanling Guo, Lang Wang, Yue’E Shen, Weisong Liu

**Affiliations:** Department of Neurology, The First Affiliated Hospital of Harbin Medical University, Harbin, Heilongjiang, China; PLOS: Public Library of Science, UNITED STATES OF AMERICA

## Abstract

**Objective:**

This study identified potential autophagy-related genes (ARGs) in atherosclerosis using bioinformatics strategies, aiming to explore novel therapeutic targets for the clinical management of atherosclerosis.

**Methods:**

Four gene expression datasets associated with atherosclerosis (GSE100927, GSE43292, GSE28829, and GSE20129) were retrieved from the GEO database of the NCBI and subjected to comprehensive analysis. A list of ARGs was compiled by accessing the HADb. AR-DEGs were screened using the “limma” package in R software. Thereafter, six core genes were identified through the combined application of three machine learning algorithms and PPI network analysis, with subsequent validation conducted using external datasets GSE28829 and GSE20129. Additional analyses included GO annotation, KEGG pathway enrichment, drug prediction, immune infiltration assessment, ceRNA network establishment, and transcription factor (TF) regulatory network analysis. Finally, the expression levels of *CASP1* in both in vivo and in vitro models were quantified using WB, RT-qPCR, and IF assays.

**Results:**

A total of 13 AR-DEGs were detected with significant expression differences between the AS group and the control group. After integrative screening via PPI network analysis and machine learning algorithms, followed by validation against external datasets GSE28829 and GSE20129, *PRKCD, SERPINA1, CASP1, CXCR4, and CCR2* were confirmed as characteristic biomarkers for AS, and their corresponding microRNAs (miRNAs) were predicted. Immune infiltration analysis indicated that these characteristic genes were correlated with immune cells. Moreover, in vivo and in vitro experimental results demonstrated that *CASP1* was highly expressed in AS samples.

**Conclusion:**

*PRKCD, SERPINA1, CASP1, CXCR4,* and *CCR2* were identified as key genes in atherosclerosis, and their associated ceRNA and TF regulatory networks were predicted. Among these key genes, *CASP1* is a promising candidate associated with atherosclerosis and worthy of further study.

## Introduction

Atherosclerosis is a long-standing inflammatory disease that causes significant alterations in the structure and function of the arterial wall [1,2]. Its onset and progression are critically influenced by endothelial dysfunction: following endothelial injury, lipid accumulation and fibrous tissue proliferation lead to plaque formation, which narrows the arterial lumen, impairs blood flow, and increases the risk of cardiovascular and cerebrovascular events [3]. In recent years, the incidence of atherosclerosis has risen markedly, posing a major threat to public health worldwide [4]. Although cholesterol-lowering therapies such as statins and PCSK9 inhibitors effectively reduce blood lipid levels and mitigate cardiovascular risk [5], atherosclerosis remains a leading cause of global mortality. Therefore, a comprehensive understanding of its pathophysiological mechanisms is essential for developing more effective diagnostic and therapeutic strategies.

Autophagy is a conserved cellular process that maintains homeostasis by degrading dysfunctional organelles and intracellular waste through lysosomal pathways [6]. This process is regulated by autophagy-related genes, with sequential activation of upstream signaling molecules. Accumulating evidence indicates that autophagy is closely associated with inflammatory responses, oxidative stress, and apoptotic processes in atherosclerosis [7–9]. In murine atherosclerosis models, silencing of autophagy-related genes (e.g., ATG5) enhances macrophage apoptosis, oxidative stress, and plaque necrosis, while promoting endothelial dysfunction [7]. Furthermore, enhanced autophagic activity protects against endothelial injury by clearing damaged organelles and reducing oxidative stress and inflammation [8–10]. However, the specific molecular mechanisms by which autophagy regulates endothelial function in atherosclerosis remain incompletely understood.

Notably, autophagy, pyroptosis, and inflammation are closely interconnected in the pathogenesis of atherosclerosis. [11] In the arterial microenvironment, inflammatory activation not only contributes to endothelial dysfunction but also modulates autophagic flux. [12] Pyroptosis, as a pro-inflammatory form of cell death, interacts with autophagy to regulate atherosclerotic lesion progression. [11] Importantly, autophagy serves as a central protective mechanism: by maintaining cellular homeostasis and mitigating inflammatory responses, it alleviates endothelial injury and retards plaque development—highlighting its pivotal role in atherosclerosis.

This study integrated bioinformatics analysis, machine learning, and experimental validation to explore the role of autophagy in atherosclerosis. Compared with conventional differential expression analysis, machine learning algorithms offer distinct advantages: they effectively reduce data noise, avoid cohort bias, and enable the robust screening of core autophagy-related biomarkers from multi-dataset transcriptomic data. [13] Using these approaches, we identified key regulatory genes and constructed a ceRNA regulatory network, while also investigating the immune landscape associated with autophagy-related genes. Additionally, we predicted therapeutic targets to provide a theoretical basis for clinical translation. Collectively, our findings shed light on the molecular mechanisms underlying atherosclerosis and offer novel insights for the development of targeted diagnostic and therapeutic strategies.

## Materials and methods

### Data collection

Two microarray datasets related to atherosclerosis, GSE100927 and GSE43292, were downloaded from the GEO database (https://www.ncbi.nlm.nih.gov/geo/). The GSE100927 dataset included 69 atherosclerotic plaque samples and 35 normal arterial tissue samples, whereas GSE43292 consisted of 32 atherosclerotic samples and 32 matched normal arterial tissue samples. Datasets GSE28829 and GSE20129 were employed as independent validation cohorts. Additionally, autophagy-related genes (ARGs) were retrieved from the HADb (http://www.autophagy.lu/).

### Identification of AR-DEGs

DEGs were identified using the “limma” package in the R programming environment. Due to inherent differences in sample size, data quality and detection platform between the two datasets, the screening criteria were defined as p < 0.05 and |log_2_FC| ≥ 1.5 for GSE100927, while for GSE43292, a slightly lower fold change cutoff of |log_2_FC| ≥ 1.2 was applied with the same statistical significance level. A total of 222 ARGs retrieved from the HADb were intersected with the identified DEGs to obtain AR-DEGs. Venn diagrams were plotted via the Sangerbox platform (http://vip.sangerbox.com/) to visualize the overlapping DEGs across datasets.

### Functional enrichment analysis

To explore the biological functions of the identified AR-DEGs in atherosclerosis, we performed GO and KEGG pathway enrichment analyses to characterize their roles in biological processes and molecular signaling pathways. GO enrichment results were further categorized into three standard functional modules: BP, CC, and MF.

### Selection of feature genes

To accurately screen hub genes, we integrated the MCC algorithm embedded in the cytoHubba plugin with three machine learning approaches for cross-validation and biomarker identification. First, a PPI network was constructed based on AR-DEGs using the STRING database (https://string-db.org/) and visualized via Cytoscape software (version 3.8.2). The cytoHubba plugin was utilized to calculate the node degree of each AR-DEG, and the top 10 genes ranked by degree score were identified as candidate hub genes.LASSO regression analysis was performed using the glmnet package in R. The penalty parameter α was set to 1, and the optimal lambda value was determined via ten-fold cross-validation to optimize model fitting. The randomForest package was adopted for RF analysis, while the e1071 package was used to conduct SVM-RFE analysis, with the radial basis function selected as the kernel function for feature gene screening. In the RF model, the number of decision trees was set to 100 to prevent overfitting, and model performance was evaluated according to classification accuracy. Ultimately, the overlapping genes screened by all four algorithms were defined as the key autophagy-related biomarkers for atherosclerosis.

### Validation of diagnostic biomarkers

The diagnostic performance of the screened biomarkers was evaluated by receiver operating characteristic (ROC) curve analysis using the pROC package in R, with the GSE28829 and GSE20129 datasets served as external validation cohorts. An area under the curve (AUC) value greater than 0.7 was defined as acceptable diagnostic efficacy.

### Immune cell infiltration analysis

Immune functional disparities between the high- and low-expression subgroups of target genes were analyzed using the R packages GSVA, GSEABase, ggpubr, and reshape2. The CIBERSORT algorithm (version 1.0) with the standard LM22 immune signature gene set was further utilized to explore the relationships between hub gene expression and the infiltration abundance of 22 immune cell subtypes.

### Construction of ceRNA and TF regulatory networks

miRmap (https://mirmap.ezlab.org), TargetScan (http://www.targetscan.org) databases, and the miRDB (https://mirdb.org) were used to predict microRNAs that potentially interact with the target gene. SpongeScan (http://spongescan.rc.ufl.edu) was applied to identify interactions between the predicted miRNAs and their upstream long non-coding RNAs (lncRNAs). TF regulatory networks were constructed using the NetworkAnalyst platform (http://www.networkanalyst.ca) [14]. The constructed networks were displayed using Cytoscape (version 3.8.2).

### Prediction of potential therapeutic drugs

The DGIDB (http://www.dgidb.org) [15] is an integrated public resource that documents potential therapeutic agents targeting specific genes. In this study, gene–drug interaction analysis was performed using DGIdb to identify candidate agents targeting the identified core genes and assess their therapeutic potential in atherosclerosis. The resulting gene–drug interaction network was visualized using Cytoscape software.

### Cell culture and in vitro model establishment

HUVECs were purchased from iCell Bioscience (Shanghai, China). Cells were cultured in high-glucose Dulbecco’s modified Eagle’s medium (DMEM; Gibco, USA) supplemented with 10% fetal bovine serum (FBS; Procell, China) and 1% penicillin–streptomycin solution (Beyotime, China), and maintained at 37 °C in a humidified atmosphere containing 5% CO_2_. To establish an in vitro atherosclerotic model, HUVECs were stimulated with 100 μg/mL oxidized low-density lipoprotein (ox-LDL; Solarbio, China) for 24 h.

### Animal model

Male C57BL/6J and ApoE^-/-^ mice (6–8 weeks old, approximately 20 g) were purchased from Saiye Biological Technology Co., Ltd. (Suzhou, China). All mice were acclimatized for 3 days before dietary intervention. ApoE^-/-^ mice were fed a high-fat diet (HFD), while wild-type (WT) C57BL/6J mice were given a standard chow diet for 12 consecutive weeks. All animals were housed under controlled conditions with a 12 h light/dark cycle, ambient temperature of 22–26 °C, and relative humidity of 40%–70%. For euthanasia, mice were subjected to cardiac perfusion with normal saline via left ventricular puncture. All animal procedures were conducted in accordance with the National Institutes of Health (NIH) guidelines and approved by the Institutional Animal Care and Use Committee (IACUC) of Harbin Medical University. Ethical approval number: 2025055.

### Cell transfection

Log-phase HUVECs were trypsinized, seeded into 6-well plates (2 mL/well), and cultured overnight at 37°C. At 60–70% confluence, siRNAs (SevenFast, Beijing, China) were transfected using jetPRIME® (Polyplus) following the manufacturer’s protocol. Transfection complexes were prepared by mixing reagent and siRNA at the specified ratio, incubated for 20 min at room temperature, and 200 µl added to each well. After 24 h at 37°C, medium was replaced and cells cultured for another 24 h before evaluating silencing efficiency. The siRNA sequences were as follows: **si-*CASP1***, 5′-3′:GCUCUUCCACACCAGAUAA(dT).(dT),3′-5′: UUAUCUGGUGUGGAAGAGC(dT) (dT).

### qRT-PCR analysis

Total RNA was extracted using an RNA isolation kit (SevenFast, Beijing, China). Complementary DNA (cDNA) was subsequently reverse-transcribed with the ReverTra Ace™ qPCR RT Kit (TOYOBO, Japan). Quantitative real-time PCR (qRT-PCR) was performed on an ABI 7500 Real-Time PCR System using SYBR Green qPCR MasterMix II (SevenFast, Beijing, China). Relative gene expression levels were quantified via the 2^-^ΔΔCt method. The sequences of the primers used are listed below: **β-actin**, forward: 5′-CACCATTGGCAATGAGCGGTTC-3′, reverse: 5′-AGGTCTTTGCGGATGTCCACGT-3′; ***CASP1***, forward: 5′-GCTGAGGTTGACATCACAGGCA-3′, reverse: 5′-TGCTGTCAGAGGTCTTGTGCTC-3′.

### WB analysis

Total proteins were extracted from HUVECs using RIPA lysis buffer, and protein concentrations were quantified with a BCA protein assay kit (Beyotime, China). Equal amounts of protein samples were separated via 7.5% SDS–PAGE and then transferred onto nitrocellulose membranes. The membranes were blocked with 5% non-fat milk for 1 h at room temperature. Subsequently, membranes were incubated overnight at 4 °C with primary antibodies against CASP1 (22915–1-AP, 1:7000 dilution; Proteintech) and β-actin (66009–1-Ig, 1:5000 dilution; Proteintech). After washing, membranes were incubated with DyLight 800-conjugated secondary antibodies (1:5000 dilution; Abbkine) for 1 h at room temperature. Protein bands were visualized and densitometrically quantified using ImageJ software.

### Immunofluorescence (IF)

After three washes with PBS, cells were fixed in 4% paraformaldehyde (PFA, pH 7.4) at room temperature for 20 min. Subsequently, cells were permeabilized with 0.2% Triton X-100 and blocked with 5% BSA for 1 h. Thereafter, cells were incubated with the primary anti-CASP1 antibody overnight at 4 °C.

### HE staining

Aortic and carotid arterial tissues were fixed in 4% paraformaldehyde, followed by routine dehydration and paraffin embedding. Serial sections (5μm in thickness) were prepared using a cryostat at an optimal cutting temperature compound. For hematoxylin-eosin (HE) staining, sections were first stained with hematoxylin for 10 min and rinsed thoroughly with tap water until nuclear bluing appeared. Sections were then counterstained with eosin for 3 min. Afterwards, the slides were sequentially immersed in a graded ethanol series (75%, 85%, 95%, and 100%) for 2–3 s per concentration, followed by complete dehydration and xylene clearing. Finally, the sections were sealed with neutral balsam, and histological images were observed and acquired under a light microscope.

### Oil red o staining

Aortic and carotid arterial tissues were harvested and fixed in 4% paraformaldehyde for 24h. The intact arterial tissues were dissected and incubated in Oil Red O working solution for 30 min, followed by thorough rinsing with PBS. Samples were then immersed in 60% isopropanol for 1 min, and nuclei were counterstained with hematoxylin for 2 min. After rinsing with tap water for 10 min until nuclear bluing was observed, the sections were mounted with neutral balsam. Finally, the formation of arterial lipid plaques was examined under a light microscope.

### Statistical analysis

Statistical analysis was performed using SPSS 24.0. All quantitative data are presented as the mean ± standard deviation (SD) from at least three independent experiments. GraphPad Prism 8.0 was used for data plotting and statistical evaluation. One-way analysis of variance (ANOVA) was adopted for comparison among multiple groups, and Student’s t-test was used for comparison between two groups. Tukey’s post hoc test was applied for pairwise comparisons among groups. A value of *P* < 0.05 was considered statistically significant (*P < 0.05; **P < 0.01; ***P < 0.001; ****P < 0.0001).

## Results

**Fig 1:**
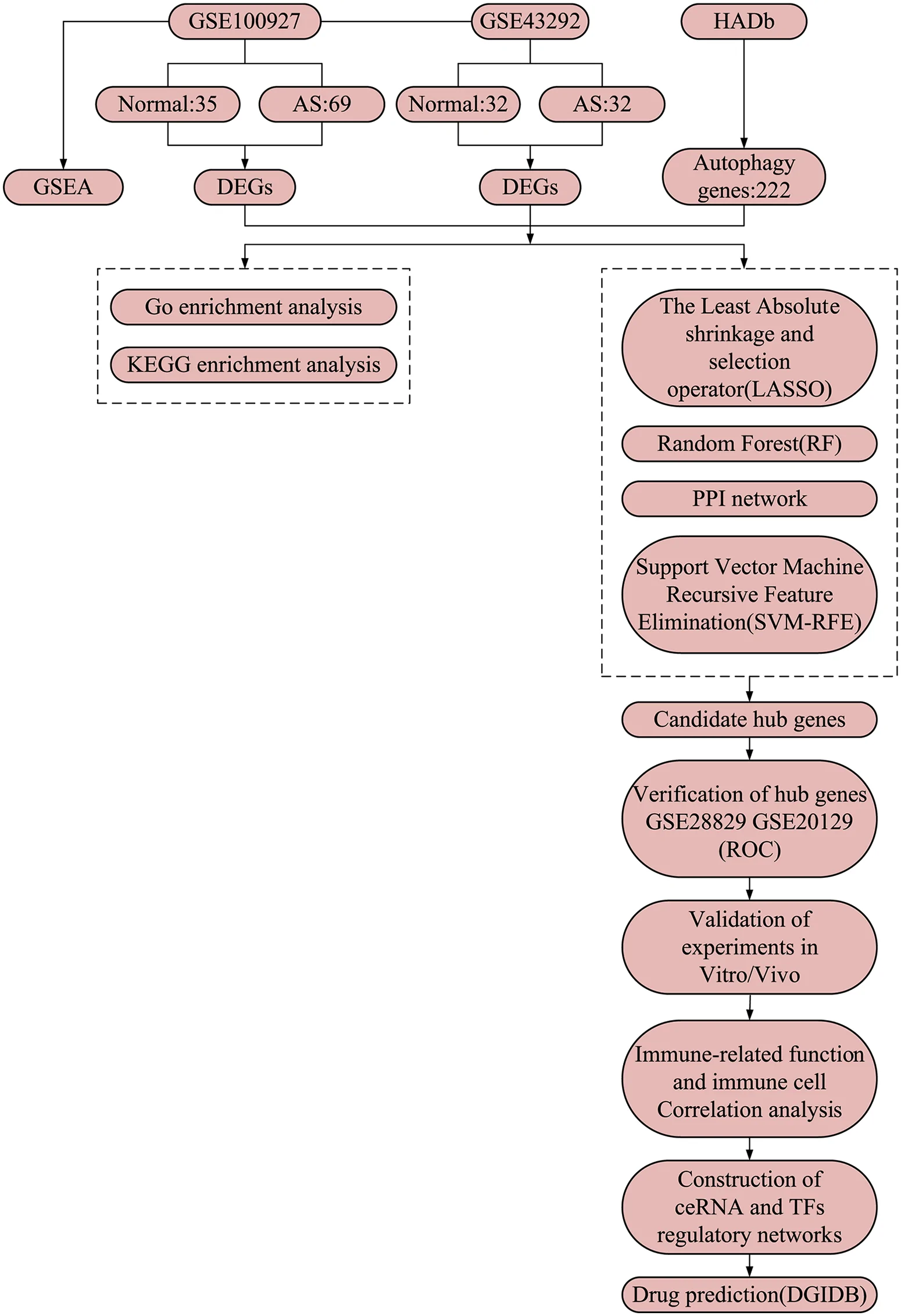
Flowchart Of The Study.

Overall workflow of this study (Fig 1).

### Screening of AR-DEGs in atherosclerosis

**Fig 2:**
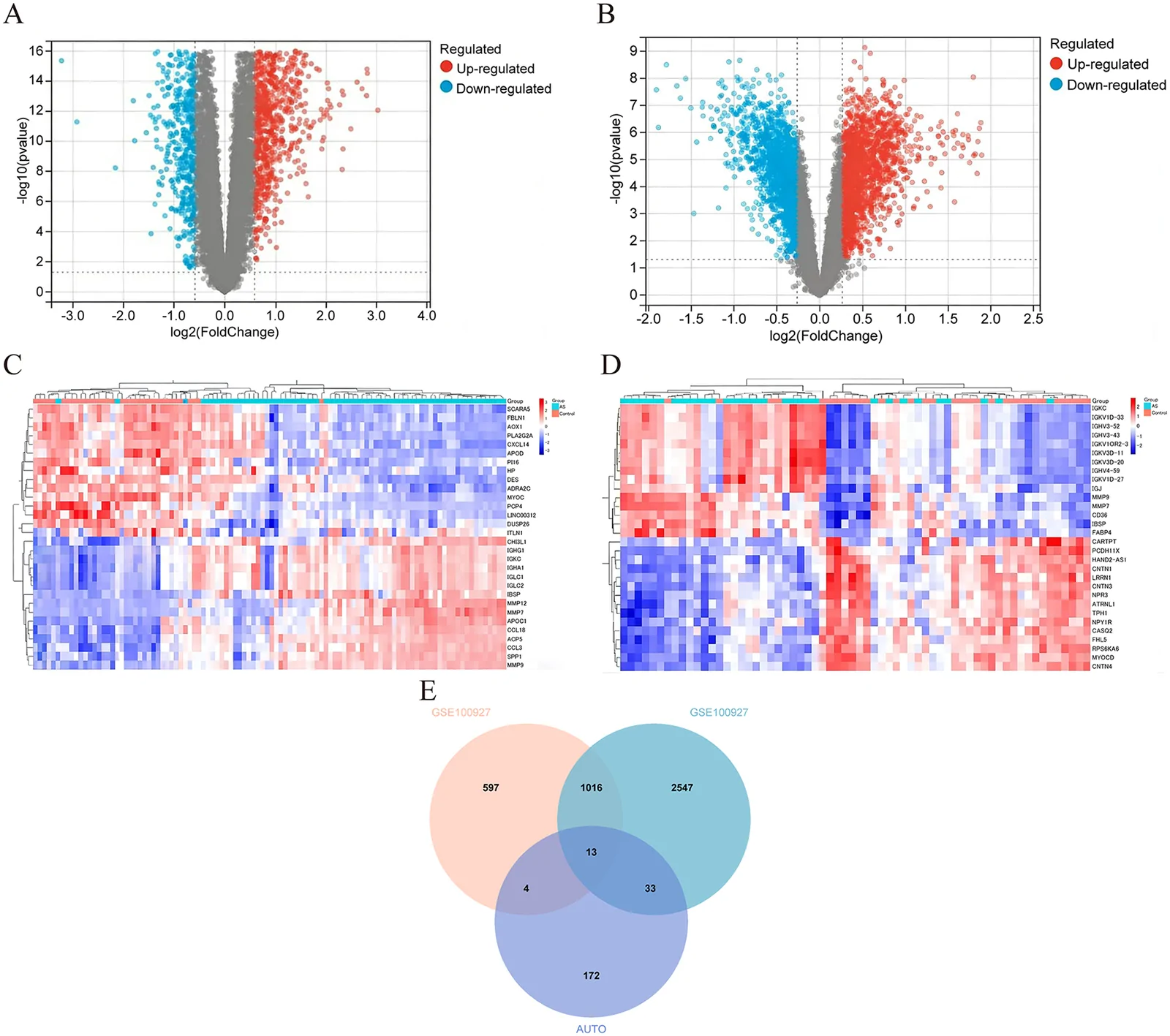
ARGs in atherosclerosis samples. **(A)** Volcano plot of GSE100927 DEGs. **(B)** Volcano plot of GSE43292 DEGs. **(C, D)** Heatmaps of top 15 up- and downregulated genes. **(E)** Venn diagram of DEGs overlap with 222 ARGs.

GSE100927 (69 AS, 35 controls) and GSE43292 (32 AS, 32 controls) were analyzed for DEGs using limma GSE100927 yielded 1,630 DEGs (1,066 up, 564 down; Fig 2A), and GSE43292 yielded 3,610 DEGs (1,906 up, 1,704 down; Fig 2B). Thirteen AR-DEGs were identified by overlapping these DEGs with 222 autophagy-related genes (Fig 2E). The 15 most prominently upregulated and downregulated genes from each dataset were visualized through heatmap analysis. (Figs 2C and 2D).

### GO and KEGG enrichment analysis of AR-DEGs

**Fig 3:**
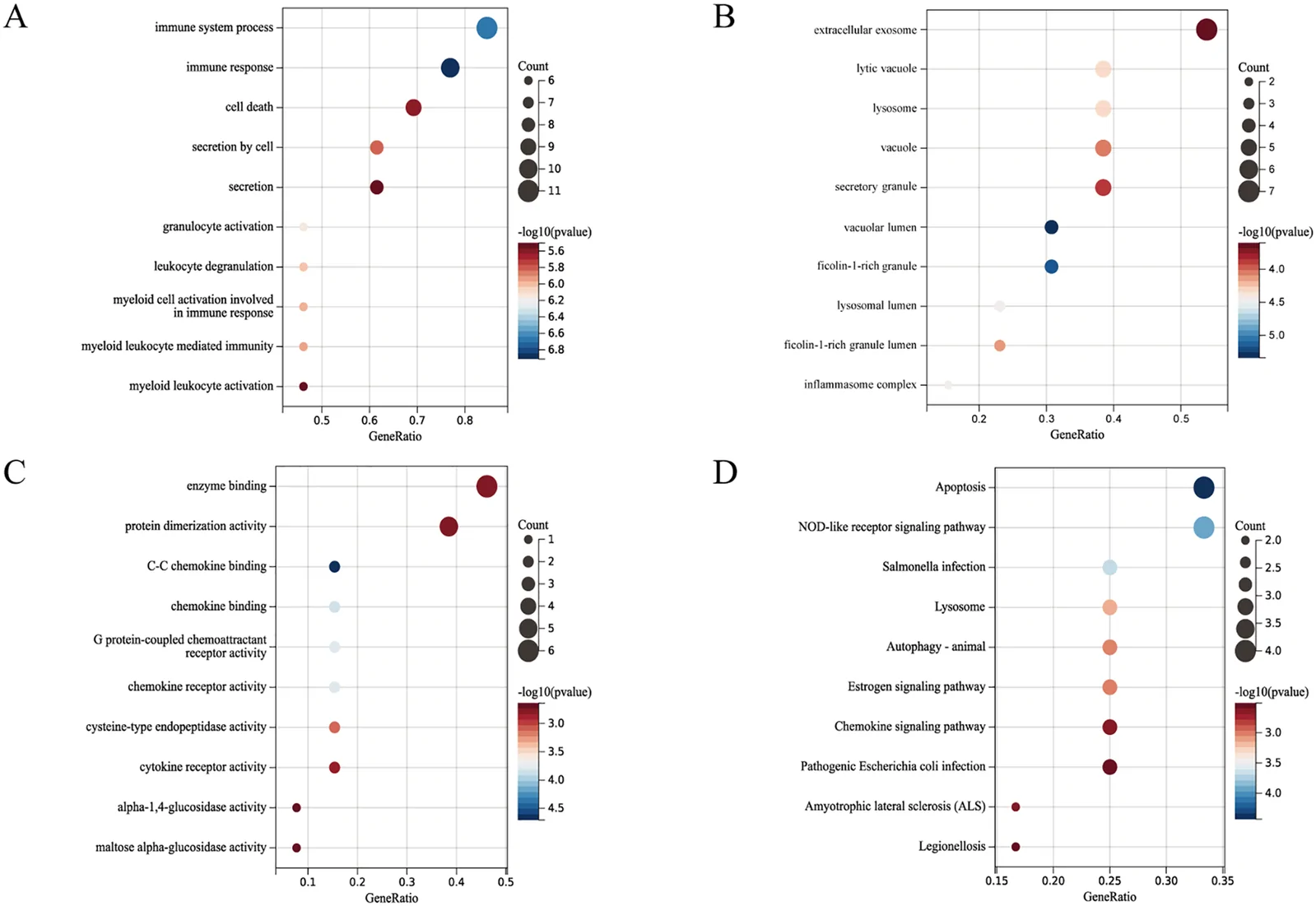
GO and KEGG enrichment analysis of AR-DEGs. **(A-C)** GO enrichment analysis. **(D)** KEGG pathway enrichment analysis.

To gain insights into the functional characteristics and molecular mechanisms underlying the AR-DEGs, enrichment analyses based on GO terms and KEGG pathways were utilized. In the BP category, AR-DEGs were primarily enriched in immune response, cell death, cellular secretion, granulocyte and myeloid cell activation, and leukocyte degranulation (Fig 3A). In the CC category, AR-DEGs were significantly enriched in extracellular exosomes, lytic vacuoles, lysosomes, secretory granules, and vacuole lumens (Fig 3B). In the MF category, AR-DEGs were enriched in enzyme binding, chemokine receptor activity, protein dimerization activity, cysteine-type endopeptidase activity, and cytokine receptor activity (Fig 3C). KEGG pathway enrichment analysis indicated that AR-DEGs were primarily involved in apoptosis, autophagy, the NOD-like receptor signaling pathway, and the chemokine signaling pathway (Fig 3D).

### Screening of hub genes

**Fig 4:**
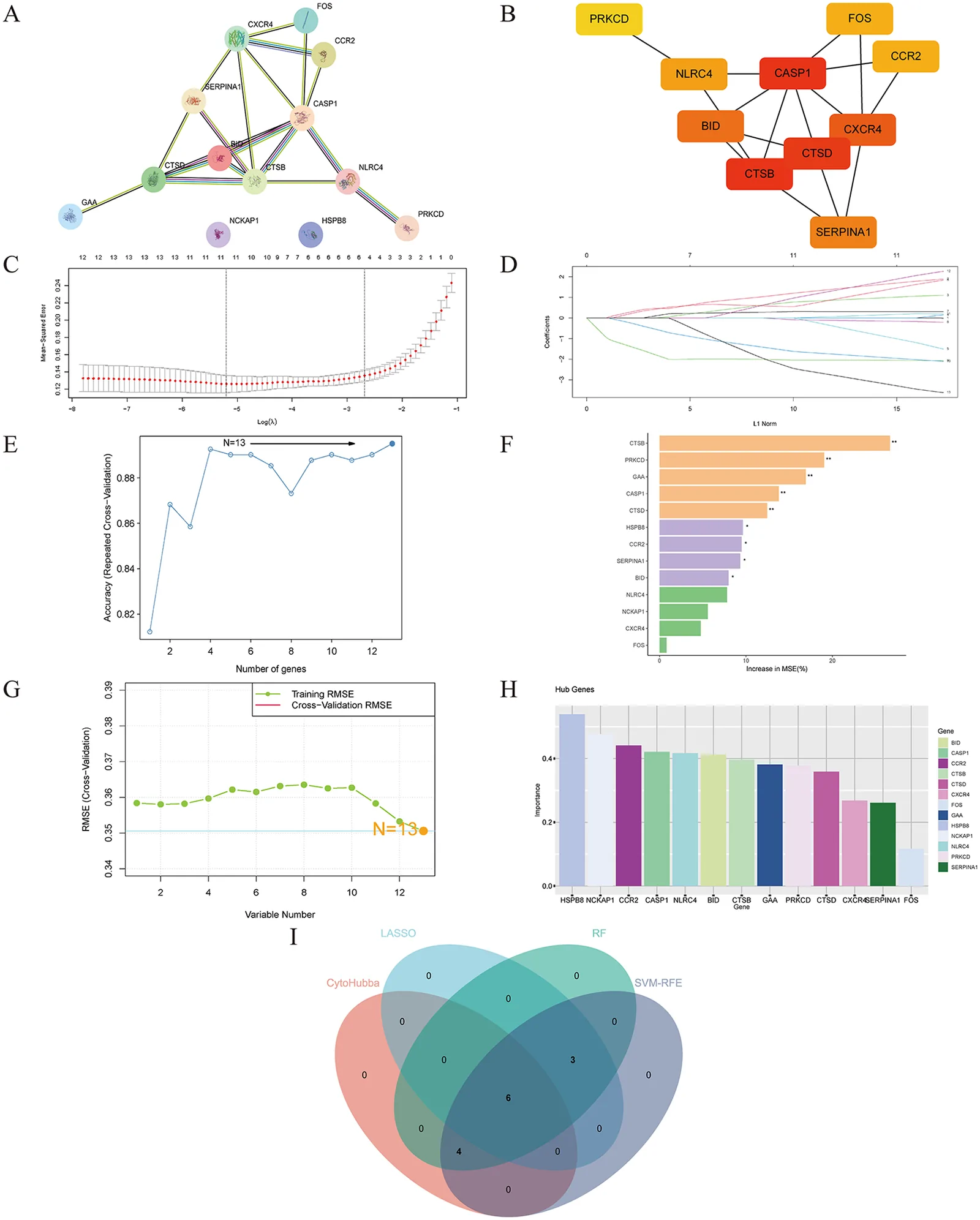
Identification of hub genes by machine learning and PPI network analysis. **(A)** PPI network constructed based on AR-DEGs. **(B)** Top 10 hub genes ranked by cytoHubba scores. **(C, D)** Cross-validation curve and LASSO coefficient profiles used for biomarker selection. **(E, F)** Feature importance ranking and selection using the RF algorithm. **(G, H)** Recursive feature elimination results and model performance based on the SVM-RFE algorithm. **(I)** Venn diagram of hub genes common to all four methods.

The 13 AR-DEGs were imported into the STRING database to construct a PPI network (Fig 4A). Then we identified the top 10 hub genes based on their node connectivity scores (Fig 4B). In the LASSO regression analysis, when the λ value was set to 0.008044378, a total of nine genes (*CASP1*, *CCR2*, *CXCR4*, *FOS*, *GAA*, *HSPB8*, *NCKAP1*, *PRKCD*, and *SERPINA1*) were selected with high accuracy (Figs 4C and 4D). In the RF analysis, genes with a relative importance greater than 1 were selected, resulting in 13 candidate hub genes (*CASP1*, *CCR2*, *CXCR4*, *FOS*, *GAA*, *HSPB8*, *NCKAP1*, *PRKCD*, *SERPINA1*, *CTSB*, *CTSD*, *BID*, and *NLRC4*) (Figs 4E and 4F). The SVM-RFE algorithm identified 13 genes when the model achieved optimal classification accuracy, which included the same 13 genes as the RF analysis (Figs 4G and 4H). Finally, six core hub genes (*PRKCD*, *CASP1*, *SERPINA1*, *CXCR4*, *FOS*, and *CCR2*) were identified by intersecting the results from all four methods and visualized using a Venn diagram (Fig 4I).

### Validation of diagnostic performance in external datasets

**Fig 5:**
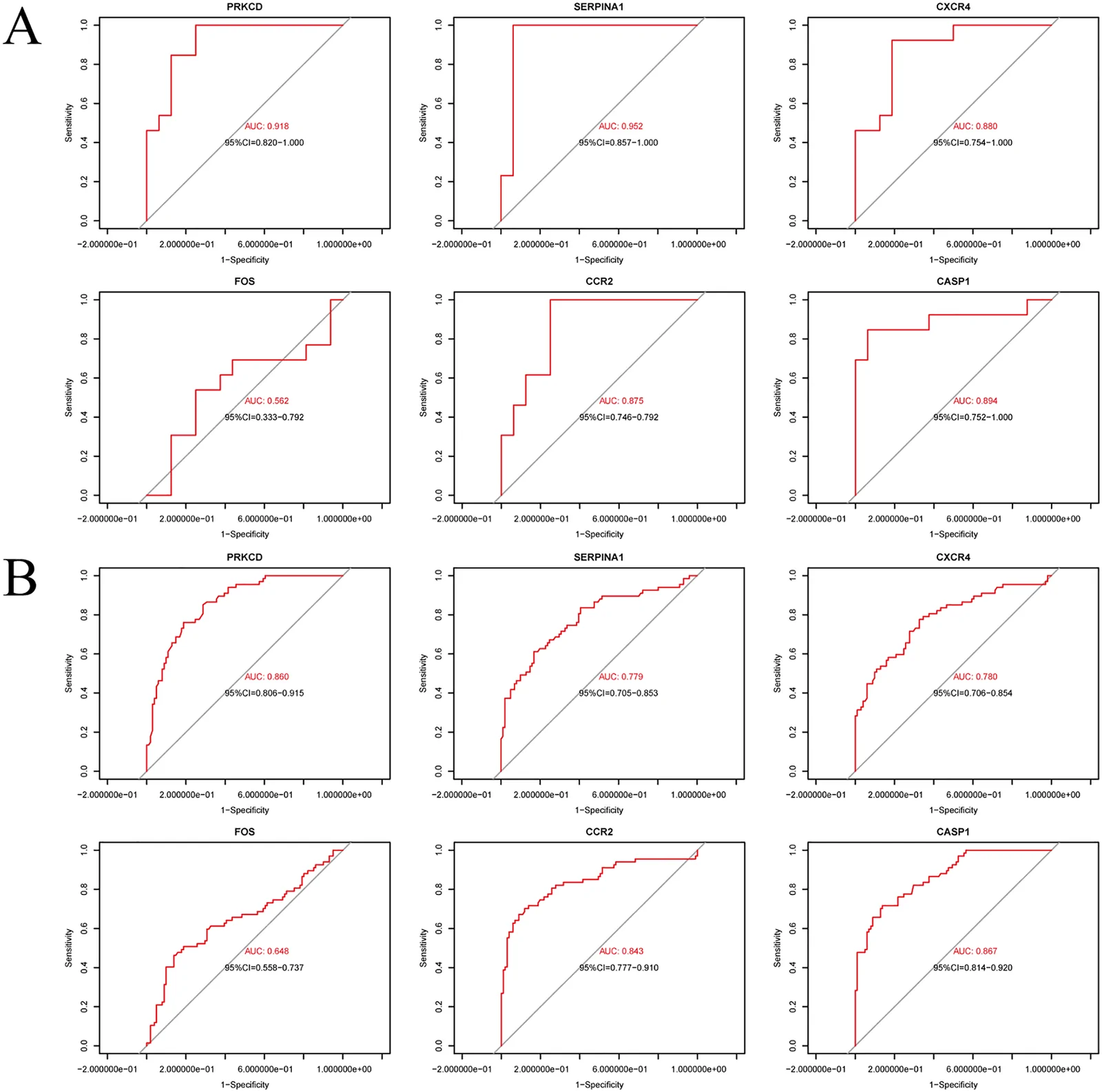
ROC curve analysis of the six diagnostic biomarkers. **(A)** Validation ROC curves. **(B)** Training ROC curves.

ROC curves were used to evaluate the diagnostic performance of the six hub genes in both validation datasets (GSE28829 and GSE20129) and training datasets (GSE100927 and GSE43292). Genes with an AUC greater than 0.7 were considered to have acceptable diagnostic value. In the validation datasets, the AUC values for *PRKCD*, *CASP1*, *SERPINA1*, *CXCR4*, *FOS*, *CCR2* were 0.918, 0.894, 0.952, 0.880, 0.562, 0.875, respectively (Fig 5A). In the training datasets, the corresponding AUC values were 0.843, 0.867, 0.779, 0.780, 0.648, and 0.843, respectively (Fig 5B). Based on these results, *PRKCD*, *CASP1*, *SERPINA1*, *CXCR4*, and *CCR2* were identified as candidate diagnostic biomarkers with favorable performance across datasets.

### Immune function and infiltration analysis of key genes

**Fig 6:**
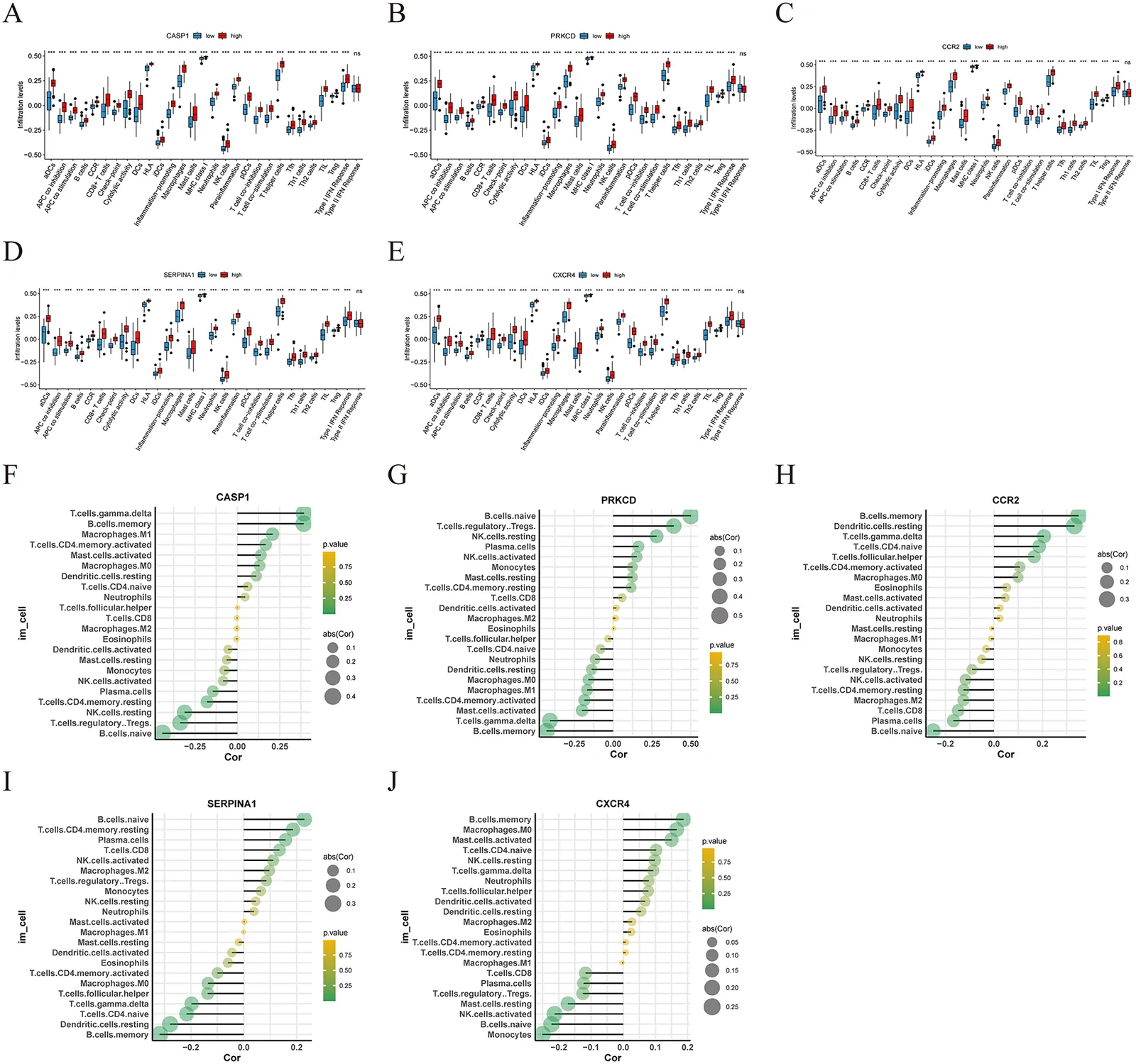
Immune-related analysis of hub genes. **(A–E)** Box plots of immune infiltration differences for the five hub genes. **(F–J)** Lollipop plots displaying the correlations between 22 immune cell types and the expression of *PRKCD*, *CASP1*, *SERPINA1*, *CXCR4*, and *CCR2*, respectively.

ssGSEA quantified immune cell infiltration across 29 subsets, and correlation analyses further characterized the associations between hub gene expression and immune infiltration profiles. High expression of the hub genes (*PRKCD*, *CASP1*, *SERPINA1*, *CXCR4*, and *CCR2*) was associated with significantly elevated infiltration levels of 28 immune cell types (Figs 6A–E). Specifically, the correlation analysis results revealed that *PRKCD*, *CASP1*, *SERPINA1*, and *CXCR4* all exhibited differential expression correlations, and *CCR2* shared this feature—its expression was positively correlated with multiple immune cells and negatively correlated with certain other cell types (Figs 6F–J).

### Construction of ceRNA and TF regulatory networks

**Fig 7:**
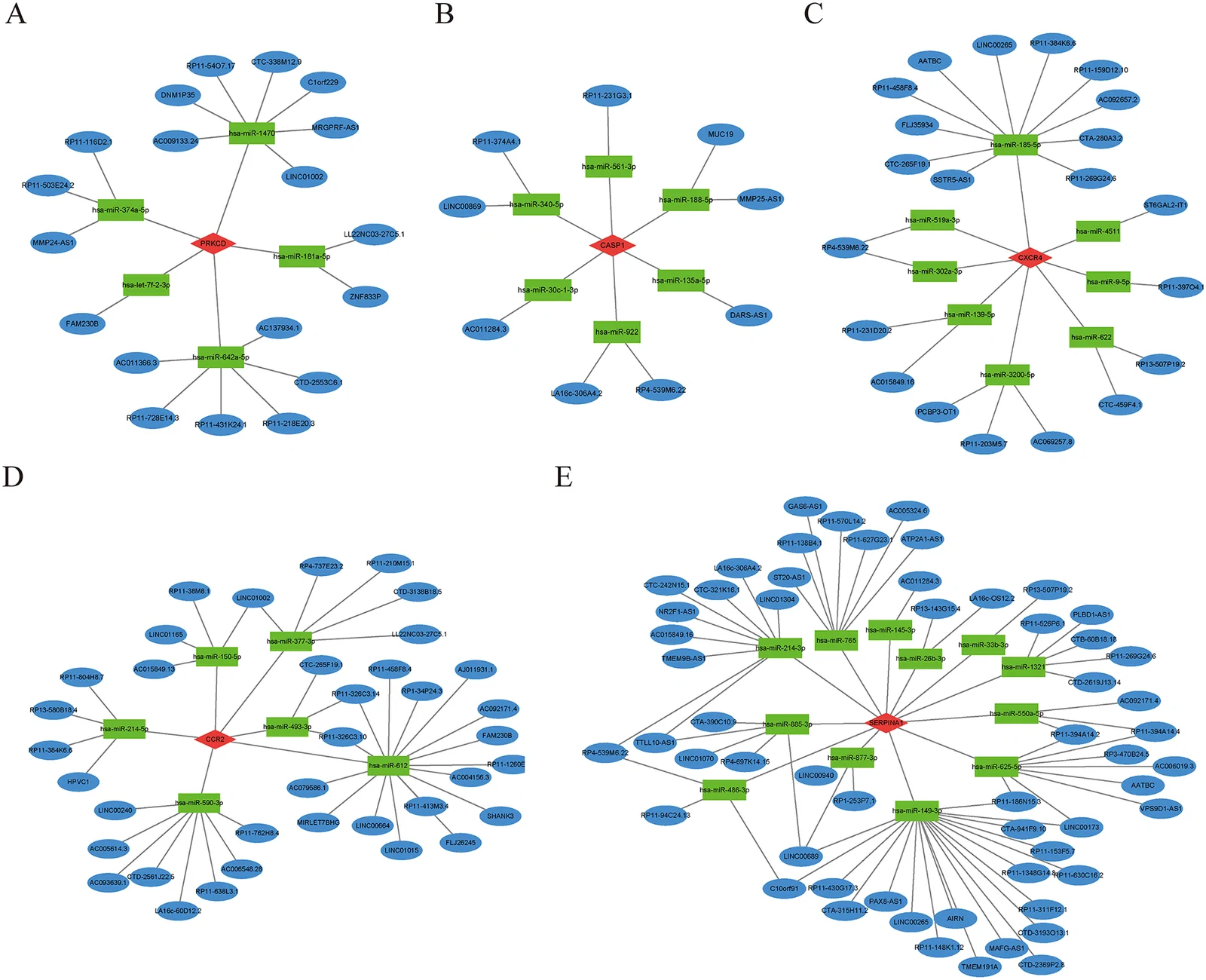
ceRNA regulatory networks of the target genes. **(A–E)** ceRNA networks constructed for the five hub genes (*PRKCD*, *CASP1*, *CXCR4*, *CCR2*, and *SERPINA1*), respectively. In the networks, Red nodes: hub genes; green nodes: miRNAs; blue nodes: lncRNAs.

To explore hub genes’ lncRNA–miRNA–mRNA regulatory mechanisms, ceRNA networks were constructed for the five selected genes. For *PRKCD*, 19 lncRNAs were predicted to interact with five targeting miRNAs (Fig 7A). Nine lncRNAs bound six miRNAs targeting *CASP1* (Fig 7B), while 21 lncRNAs interacted with eight *CXCR4*-targeting miRNAs (Fig 7C). Thirty-seven lncRNAs were associated with six *CCR2*-targeting miRNAs (Fig 7D). For *SERPINA1*, 57 lncRNAs interacted with 12 targeting miRNAs (Fig 7E).

**Fig 8:**
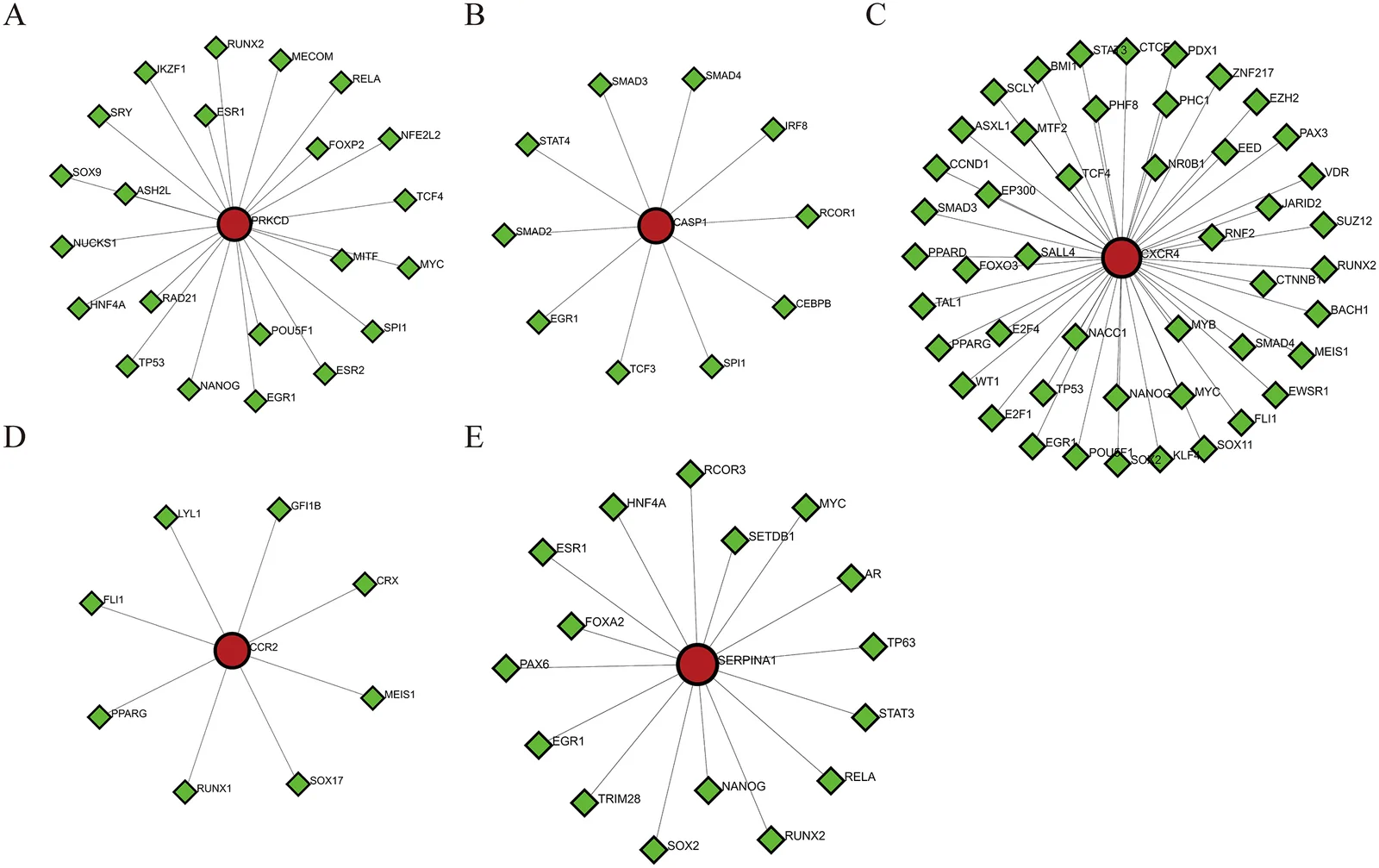
Construction of TF regulatory networks for the target genes. TF predicted to regulate the five hub genes (*PRKCD*, *CASP1*, *CXCR4*, *CCR2*, and *SERPINA1*) were identified and visualized to illustrate their potential regulatory interactions.

TFs predicted to regulate the hub genes were identified using the NetworkAnalyst platform. Specifically, 22 TFs were identified for *PRKCD*, 10 for *CASP1*, 47 for *CXCR4*, 8 for *CCR2*, and 16 for *SERPINA1* (Figs 8A-E).

### DGIdb

**Fig 9:**
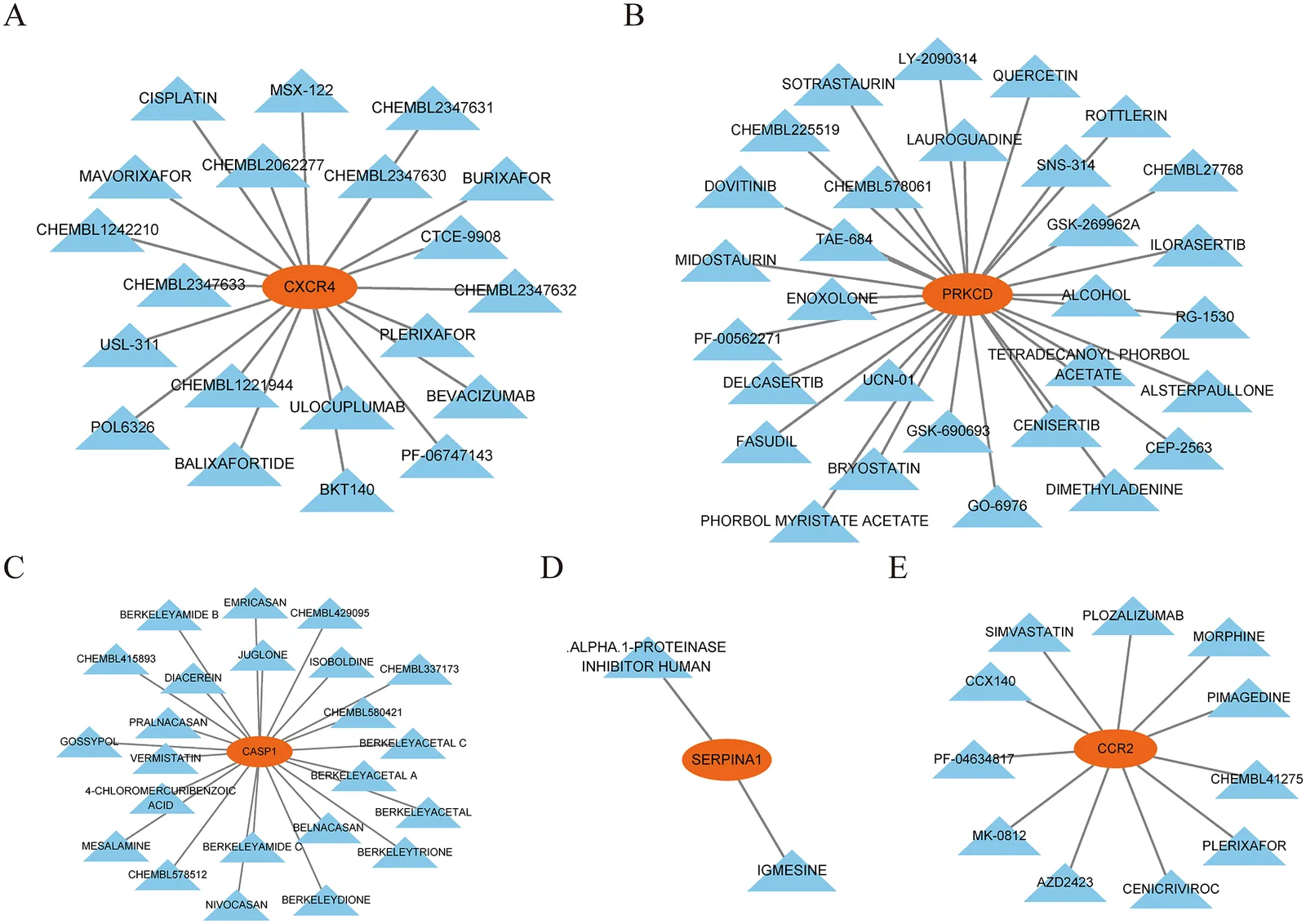
Construction of drug–gene interaction networks for the predicted therapeutic targets. **(A–E)** Drug–gene interaction networks for CXCR4, PRKCD, CASP1, SERPINA1, and CCR2, respectively.

DGIdb was used to identify potential therapeutic agents targeting the hub genes. A total of 20 drugs were predicted to target *CXCR4* (Fig 9A), 30 drugs targeted *PRKCD* (Fig 9B), 23 drugs targeted *CASP1* (Fig 9C), 2 drugs targeted *SERPINA1* (Fig 9D), and 11 drugs targeted *CCR2* (Fig 9E). These drugs may serve as potential therapeutic agents for atherosclerosis and warrant further investigation in future studies.

### Establishment of an endothelial cell injury model of atherosclerosis

**Fig 10:**
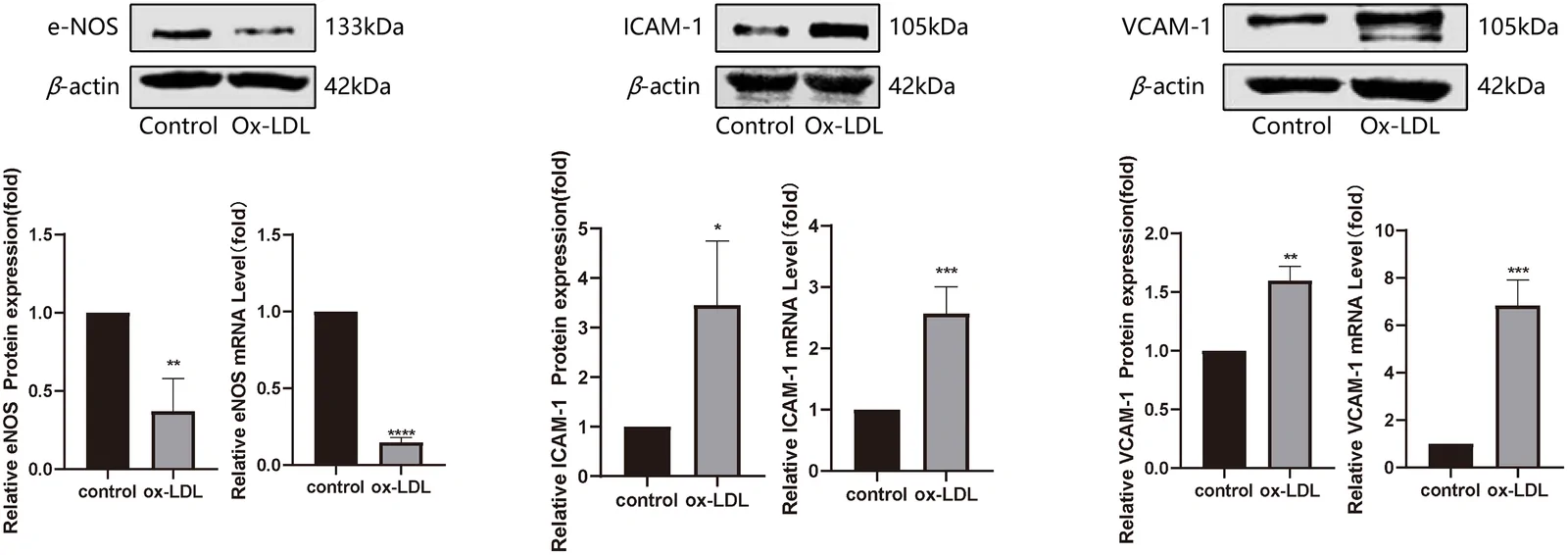
Expression of ICAM-1, VCAM-1, and eNOS in HUVECs after ox-LDL treatment for 24 h. *P < 0.05; **P < 0.01; ***P < 0.001 vs. control, n = 3.

HUVECs were treated with 100 μg/mL ox-LDL for 24 h, and endothelial injury-related markers were examined by qPCR and WB. Ox-LDL significantly increased ICAM-1 and VCAM-1 protein levels while decreasing eNOS expression (P < 0.05). Concordant mRNA changes were confirmed by qPCR (P < 0.01) (Fig 10).

### ox-LDL stimulation leads to increased *CASP1* expression in HUVECs

**Fig 11:**
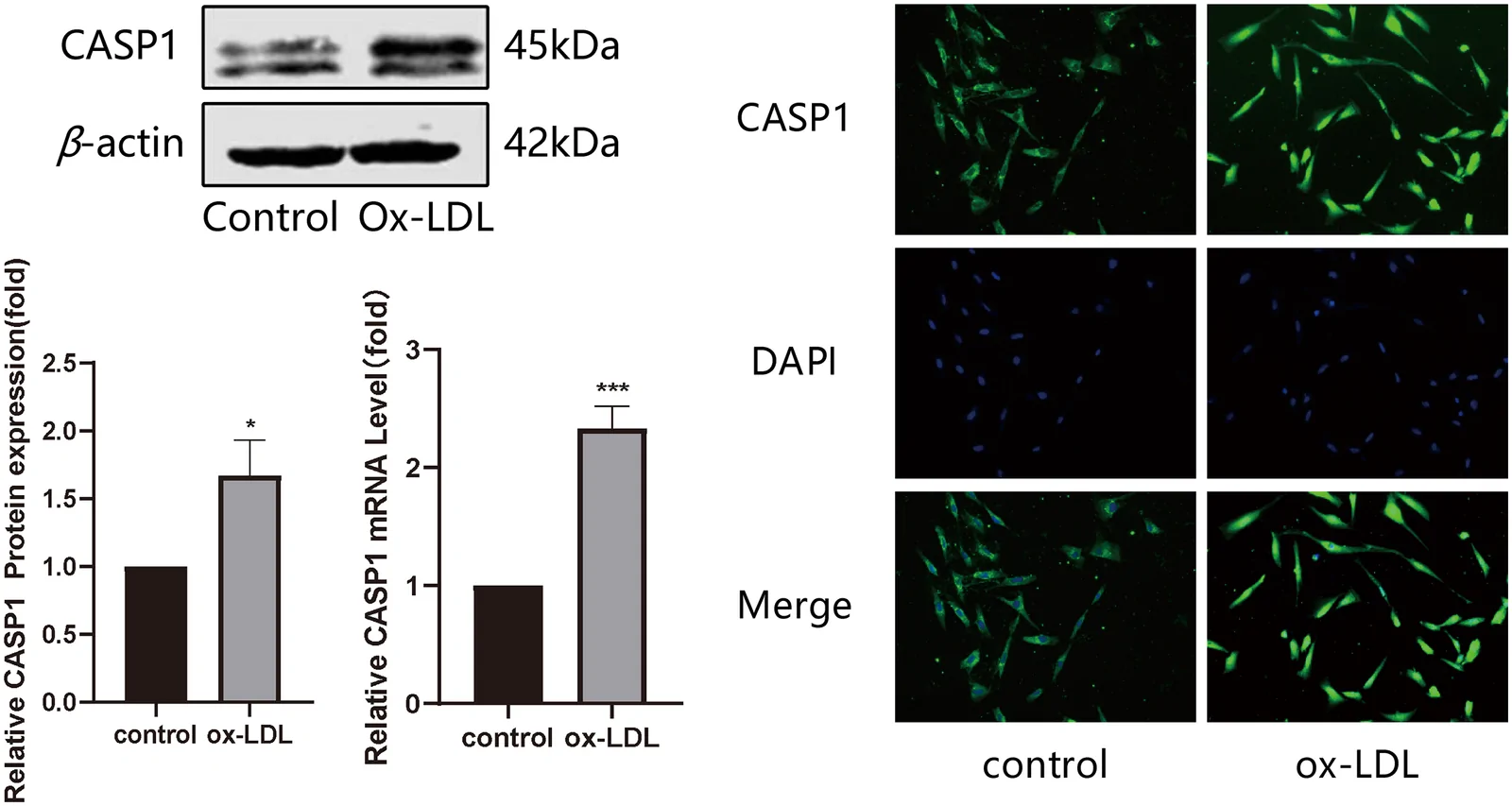
CASP1 expression in ox-LDL-induced endothelial cell injury. CASP1 protein, mRNA, and fluorescence levels were detected in ox-LDL-stimulated HUVECs. *P < 0.05; ***P < 0.001 vs. control n = 3.

We further analyzed the expression of *CASP1* in HUVECs treated with 100 μg/mL ox-LDL. Results from qPCR, Western blot, and immunofluorescence assays demonstrated that *CASP1* expression was significantly increased in endothelial cells after 24 hours of ox-LDL treatment (P < 0.01). (Fig 11)

### Silencing *CASP1* mitigated ox-LDL-induced HUVEC damage

**Fig 12:**
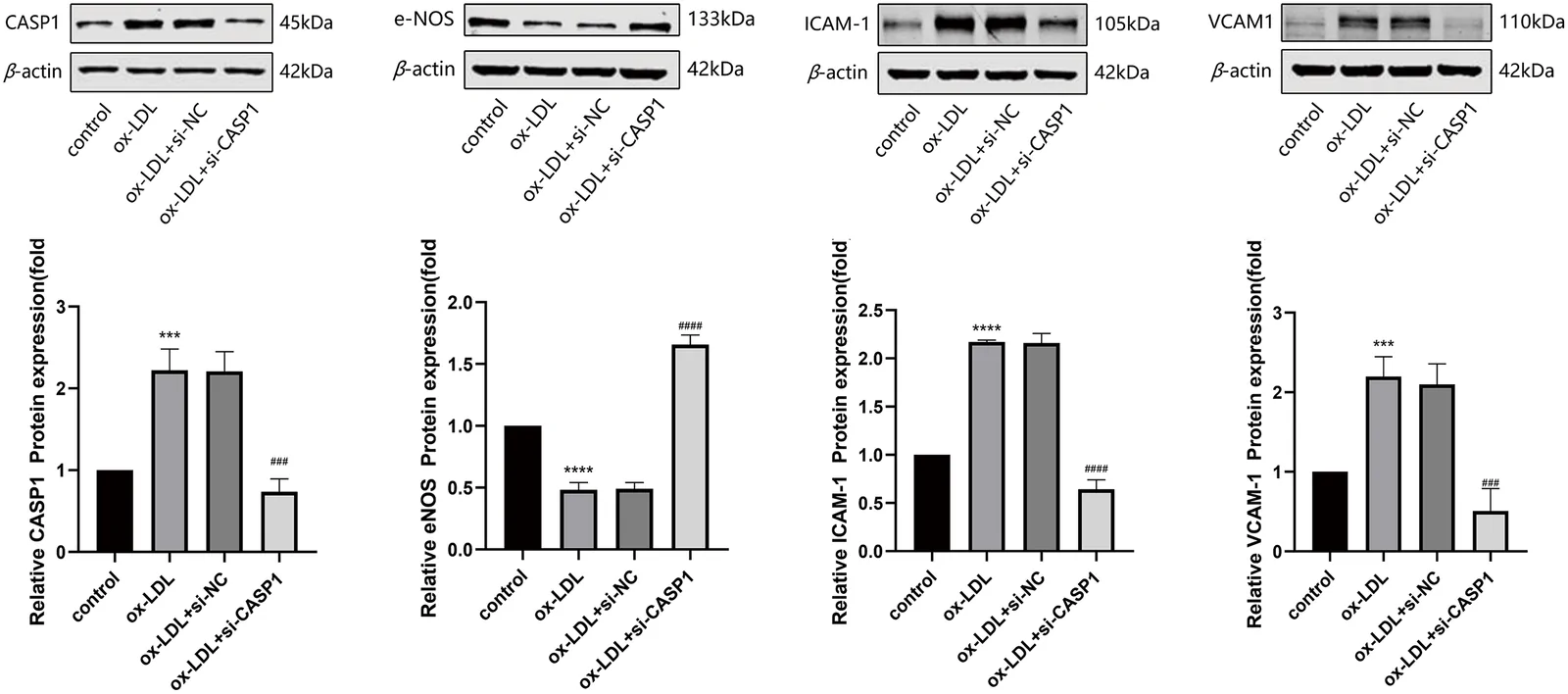
Effect of CASP1 inhibition on ox-LDL-induced endothelial injury. WB analysis of eNOS, VCAM-1, and ICAM-1 expression in CASP1-silenced HUVECs. ***P < 0.001, **P < 0.0001 vs. control n = 3, ###P < 0.001, ####P < 0.0001 vs. ox-LDL + si-NC n = 3.

After transfection with si-*CASP1*, the expression of *CASP1* and endothelial injury markers was examined by Western blot. Relative to siNC, si-*CASP1* potently suppressed ox-LDL-induced *CASP1* upregulation and abrogated the concomitant reduction in eNOS expression and induction of ICAM-1 and VCAM-1 (P < 0.001). These results indicate that inhibition of *CASP1* can ameliorate ox-LDL-induced endothelial cell injury. (Fig 12)

### Autophagy level decreases in ox-LDL-induced HUVECs injury

**Fig 13:**
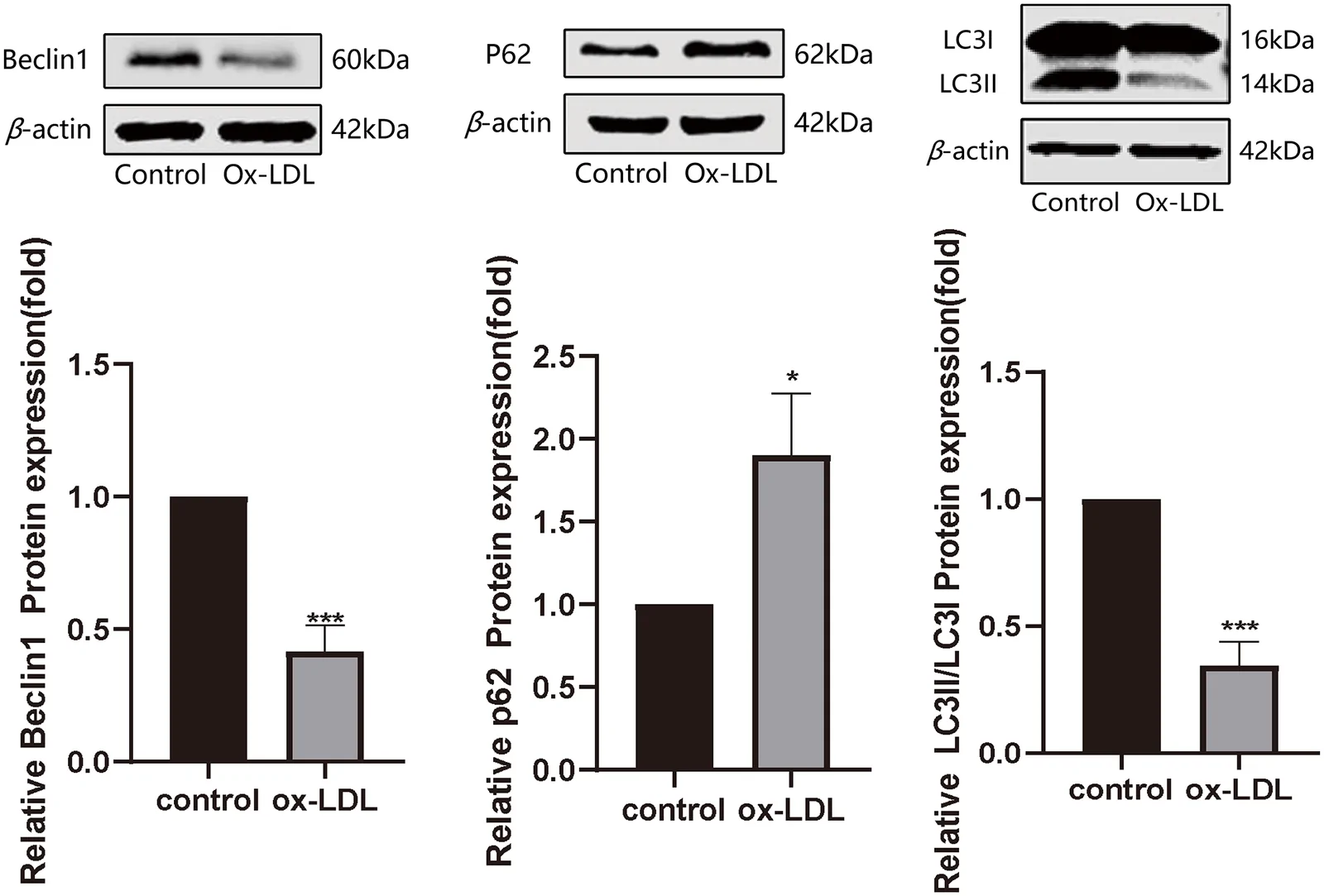
Effect of ox-LDL-Induced HUVECs Injury on Autophagy Levels. After ox-LDL-induced injury in HUVECs, the expression levels of autophagy-related proteins LC3II/I ratio, Beclin1, and p62 were detected. Compared with the control group, *p < 0.05, ***p < 0.001, n = 3.

To explore the changes of autophagy in HUVECs after ox-LDL-induced injury, HUVECs were treated with 100 μg/mL ox-LDL for 24 h. The expression levels of autophagy-related proteins LC3II/I, p62 and Beclin1 were detected by Western blot. As shown in Fig 13, compared with the Control group, the LC3II/I ratio and Beclin1 protein expression were significantly decreased (*p* < 0.001), while p62 protein expression was increased (*p* < 0.05). The experimental results indicated that ox-LDL treatment inhibited autophagy in HUVECs.

### Silencing of *CASP1* improves autophagy in ox-LDL-induced HUVECs

**Fig 14:**
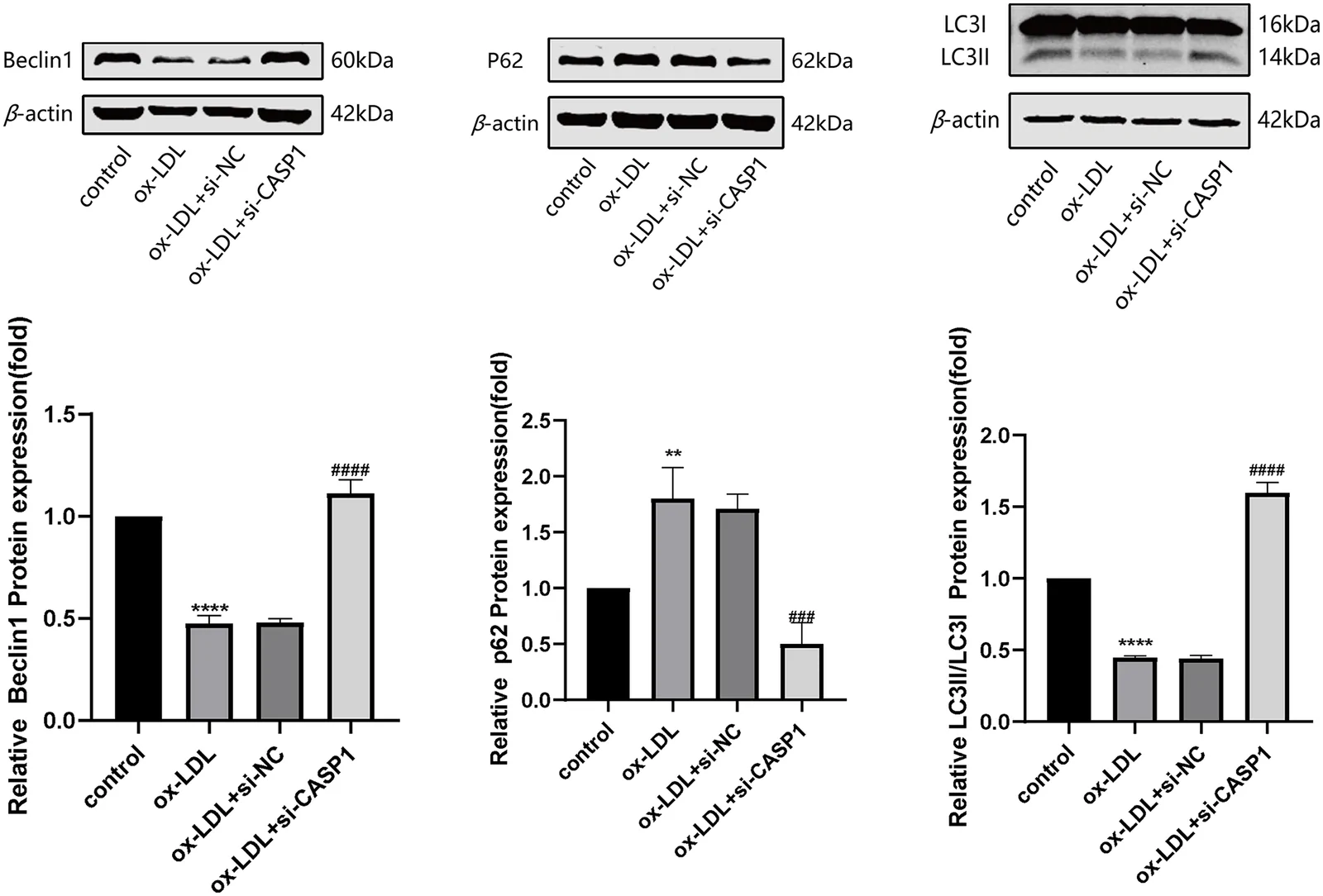
Effect of CASP1 Knockdown on Autophagy-Related Proteins in ox-LDLStimulated HUVECs. After silencing si-CASP1, the expression levels of LC3II/I, Beclin1, and p62 proteins, as well as immunofluorescence, were detected in ox-LDL-stimulated HUVECs. Compared with the Control group, **p < 0.01, ****p < 0.0001; compared with the ox-LDL + si-NC group, ###p < 0.001, ####p < 0.0001, n = 3.

We further investigated the regulatory effect of *CASP1* on autophagy in ox-LDL-treated HUVECs. As shown in Fig 14, Western blot results demonstrated that compared with the si-NC group, *CASP1* silencing reversed the ox-LDL-induced decrease in the expression of autophagy-related proteins LC3II/I and Beclin1 (*p* < 0.0001), and reversed the upregulation of p62 (*p* < 0.001). These findings indicated that silencing *CASP1* could restore impaired autophagy in ox-LDL-injured HUVECs.

### Establishment of an atherosclerotic animal model

**Fig 15:**
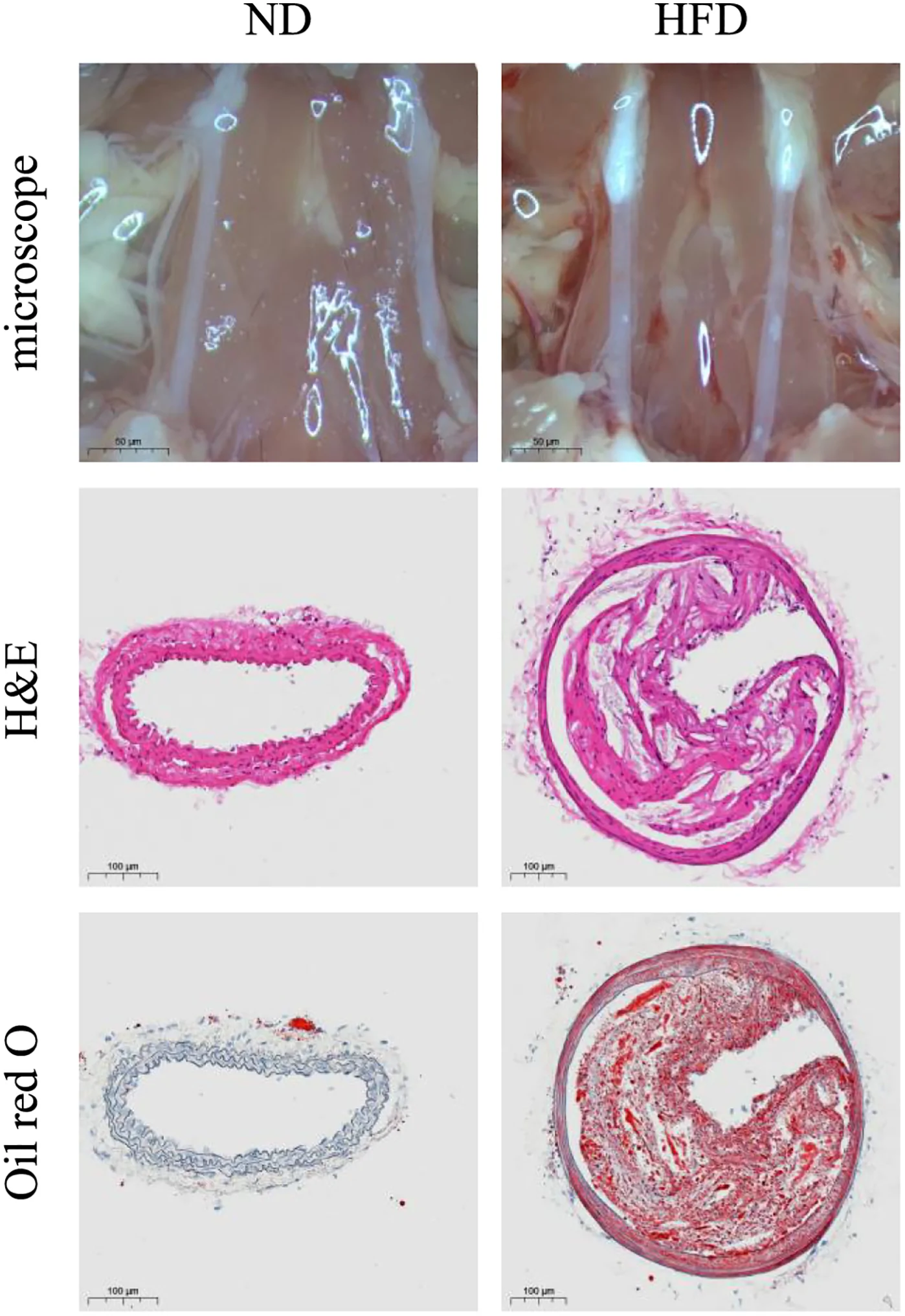
Effect of HFD on plaque area in the carotid artery and aortic root of ApoE^-/-^ mice. Representative HE and Oil Red O staining images with quantitative analysis of plaque area. Control: ApoE ^-/-^ mice fed a normal diet; HFD: ApoE^-/-^ mice fed a high-fat diet. (n = 6).

After 12 weeks of HFD feeding, ApoE^-/-^ mice were analyzed for atherosclerotic lesions in the carotid arteries and aortic roots using HE and Oil Red O staining. Compared with mice on a normal diet, HFD-fed ApoE^-/-^ mice exhibited significantly increased plaque area in both the carotid arteries and aortic roots. These results indicate that HFD feeding successfully induced atherosclerosis in ApoE^-/-^ mice. (Fig 15)

### *CASP1* expression was increased in the aortic tissues of AS mouse models

**Fig 16:**
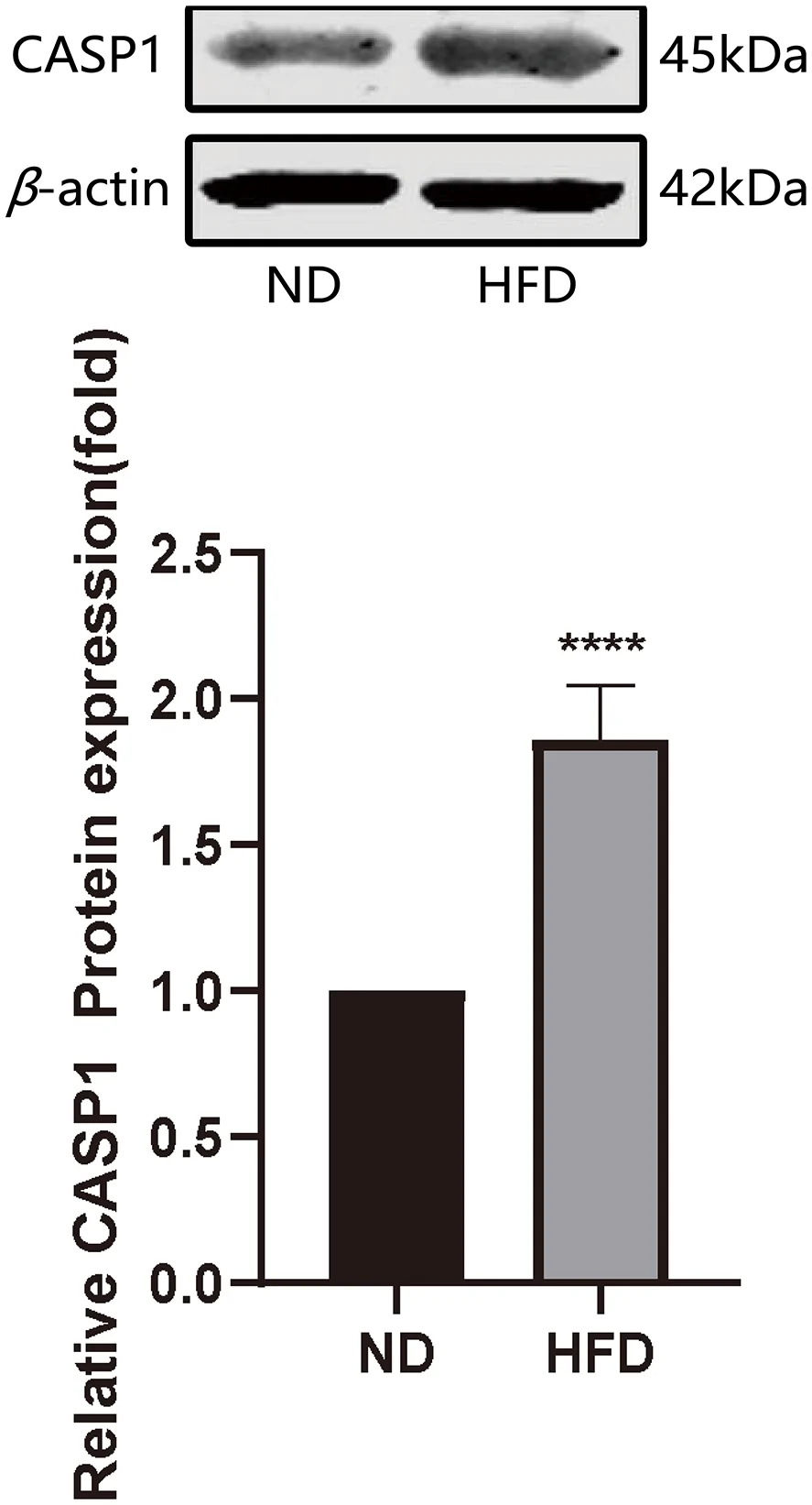
Expression Changes of CASP1 in the Aortic Tissue of ApoE^-/-^ Mice Fed a HFD. Protein expression of CASP1 in the aorta of ApoE ^-/-^ mice fed a HFD. (n = 6, ****p < 0.0001).

After establishing the AS model by feeding ApoE^-/-^ mice a HFD, WB analysis was performed to detect *CASP1* expression. Compared with mice on a normal diet, *CASP1* expression was significantly increased in HFD-fed ApoE^-/-^ mice (p < 0.0001). (Fig 16)

## Discussion

Atherosclerosis is a complex disorder that drives major adverse cardiovascular events. [16]. Common risk factors for atherosclerosis include aging, hypertension, hyperglycemia, and hyperlipidemia [17–19]. Autophagy, a key protective mechanism, plays a regulatory role in cytokine secretion by immune cells [20]. Increasing evidence suggests that modulation of autophagy can attenuate immune-mediated inflammation in vascular endothelial cells, thereby slowing the progression of atherosclerosis [21]. Thus, identifying autophagy-related hub genes in atherosclerosis may improve mechanistic understanding and aid in discovering novel therapeutic targets.

By analyzing the GSE100927 and GSE43292 datasets in combination with a predefined autophagy-related gene set, we identified a total of 13 AR-DEGs in this study. GO analysis revealed that AR-DEGs were mainly involved in apoptosis, immune response, chemokine receptor signaling, and lysosome-related functions. KEGG analysis indicated that they were implicated in autophagy, NOD-like receptor signaling, and other inflammatory pathways, all of which were closely associated with atherosclerosis.

In this study, six hub genes—*PRKCD*, *CASP1*, *SERPINA1*, *CXCR4*, *FOS*, and *CCR2*—were identified through integrated analyses combining PPI network construction, LASSO regression, RF, and SVM-RFE. ROC curve analysis revealed that, except for FOS (AUC < 0.7), the other five genes exhibited favorable diagnostic performance with AUC values > 0.7 in both the training and validation datasets, among which CASP1 presented the highest diagnostic efficiency in the validation dataset. Furthermore, the upregulation of *CASP1* was confirmed by both Western blot and qRT-PCR in ox-LDL-treated HUVECs, supporting its involvement in atherosclerosis.

*PRKCD* (protein kinase C delta), a novel PKC isoform, regulates proliferation, apoptosis, inflammation, and mitochondrial function, all of which are implicated in atherosclerosis pathogenesis [22,23]. Leitges et al. demonstrated that *PKCδ*-deficient mice developed more severe atherosclerosis compared to wild-type controls [24]. In addition, *PKCδ* inhibitors have been shown to reduce vascular inflammation by downregulating COX-2 expression, further implicating *PKCδ* in atherosclerotic regulation [25].

*CASP1* (caspase-1) is a cysteine protease involved in pyroptosis and inflammatory cytokine activation [26]. Previous studies have demonstrated that, in response to inflammatory stimuli, caspase-1 activation facilitates the processing and maturation of IL-1β and IL-18 [27]. Both IL-1β and IL-18 are closely associated with the progression of atherosclerosis [12]. IL-1β promotes the upregulation of vascular adhesion molecules, including VCAM-1 and ICAM-1, thereby contributing to endothelial dysfunction and the development of atherosclerotic lesions [28,29]. Notably, IL-1β deficiency reduces plaque burden in ApoE^-/-^ mice [29]. Activation of the caspase-1/IL-18 signaling axis is strongly correlated with atherosclerotic plaque progression. [30]. The results provide evidence for the involvement of *CASP1* in promoting atherogenesis.

The *SERPINA1* gene encodes α1-antitrypsin (AAT), an acute-phase protein produced in the liver and classified as a member of the serpin (serine protease inhibitor) family. AAT is known to regulate inflammatory responses and immune homeostasis [31]. Some studies have suggested that AAT inhibits elastase-mediated extracellular matrix degradation, thereby preserving elastic fibers in arterial walls and potentially delaying atherosclerotic progression [32]. Additionally, decreased *SERPINA1*/AAT levels have been associated with increased cardiovascular risk [33]. However, the exact role of *SERPINA1* in atherosclerosis remains unclear and warrants further investigation.

*CXCR4* is a G protein–coupled receptor that specifically binds to the chemokine *CXCL12*. It plays a critical role in maintaining vascular integrity by regulating the biological activity of endothelial cells. [34]. Studies have demonstrated that CXCR4 deficiency alleviates inflammatory responses and enhances post-ischemic angiogenic capacity [35]. Döring et al. reported that CXCR4 maintains vasoconstrictive function and vascular stability, thereby delaying the progression of atherosclerosis [36]. Furthermore, Merckelbach et al. observed significant upregulation of *CXCR4* and *CXCL12* mRNA and protein levels in human carotid atherosclerotic plaques, supporting their involvement in disease pathogenesis.

*CCR2*, the receptor for *CCL2* (MCP-1), is predominantly expressed on inflammatory monocytes and plays a key role in atherosclerosis. Boring et al. demonstrated that *CCR2* deficiency reduces lesion formation in apoE ^-^/^-^ mice [37]. Therefore, targeting *CCR2* may represent a promising therapeutic strategy to attenuate atherosclerotic progression.

Immune cell infiltration and persistent inflammatory activation are the core pathological hallmarks of atherosclerosis. Therefore, we applied the ssGSEA algorithm to systematically evaluate the immune infiltration landscape associated with these signature genes. The results showed that significant immune cell enrichment was observed in the high-expression group of signature genes, suggesting that aberrant expression of these core genes is closely linked to the remodeling of the arterial immune microenvironment. Recruited macrophages, neutrophils, and T helper cell subsets can further induce endothelial dysfunction, amplify inflammatory cascades, and synergize with pyroptosis and autophagy to jointly facilitate the initiation and progression of atherosclerotic plaques. These findings provide an important theoretical basis for our subsequent in-depth exploration of immune-related pathological mechanisms underlying atherosclerosis.

miR-188-5p is a small non-coding RNA that profoundly affects vascular endothelial cell functions by regulating cell proliferation, migration and survival. Previous studies have demonstrated that silencing of miR-188-5p can alleviate neuronal apoptosis and neuroinflammation by upregulating Lin28a expression. [38] In addition, overexpression of miR-188-5p has been shown to relieve focal cerebral ischemia/reperfusion injury in rats. [39] In the present study, by constructing ceRNA and transcriptional factor regulatory networks, we identified miR-188-5p as one of the critical upstream regulatory factors of CASP1. These findings provide novel insights and promising therapeutic targets for the treatment of atherosclerosis and related vascular diseases.

In the clinical management of AS, although statins have been widely used, the onset and progression of AS cannot be effectively controlled. In this study, the DSigDB database was employed to screen potential therapeutic agents for atherosclerosis. Several promising candidate compounds were identified, including juglone, diacerein, mesalamine, gossypol, and vermistatin. These findings provide novel candidate drugs and potential intervention strategies for the clinical prevention and treatment of atherosclerosis.

In this study, both in vitro and in vivo experiments confirmed that CASP1 expression was significantly upregulated in the atherosclerosis model. In subsequent in vitro experiments, small interfering RNA (siRNA) was used to successfully knock down CASP1, and the knockdown efficiency was verified by qPCR and Western blot. Treatment with 100 μg/mL ox-LDL induced evident endothelial cell injury. However, CASP1 silencing effectively ameliorated ox-LDL-triggered endothelial damage, reversed the downregulation of eNOS, and inhibited the increased expression of ICAM-1 and VCAM-1. These findings suggest that CASP1 serves as a pivotal biomarker for atherosclerosis (AS), which is consistent with our bioinformatics predictions and further highlights the central role of CASP1 in the pathogenesis of AS.

Accumulated studies have demonstrated that p62 binds to ubiquitinated proteins and forms complexes with the autophagosome marker LC3II, while Beclin1 is critically involved in autophagosome formation and maturation. In the present study, the LC3Ⅱ/Ⅰ ratio and p62 protein level were detected to evaluate cellular autophagic activity. The results showed that ox-LDL treatment reduced the LC3Ⅱ/Ⅰ ratio and Beclin1 expression, elevated p62 level, and ultimately suppressed autophagic function. In contrast, CASP1 knockdown reversed these abnormal molecular changes and restored impaired autophagy. Collectively, our experimental results confirm that CASP1 alleviates endothelial injury in atherosclerosis by regulating autophagy. This study clarifies the molecular mechanism underlying CASP1 function and provides a promising therapeutic target for the clinical prevention and treatment of atherosclerosis.

In conclusion, this study confirms that CASP1 delays the progression of atherosclerosis by regulating autophagy. Accordingly, CASP1 silencing is regarded as a promising intervention strategy to activate autophagy and alleviate vascular endothelial cell injury. Nevertheless, several limitations still exist in the present study. Although we have verified the role of CASP1 in endothelial cell injury during atherosclerosis through bioinformatics analysis as well as in vitro and in vivo experiments, the predicted upstream microRNAs and candidate therapeutic drugs remain to be validated by further extensive experiments. Moreover, this study is only limited to mechanistic exploration at the molecular and genetic levels, without validation using clinical samples. In future research, we will address these limitations by conducting clinical sample verification and in-depth mechanistic experiments, aiming to further elucidate the molecular pathway by which CASP1 regulates autophagy in atherosclerosis and provide more solid experimental evidence for its clinical translational application.

## Conclusion

In summary, our study identified five autophagy-related genes associated with atherosclerosis (*PRKCD, SERPINA1, CASP1, CXCR4*, and *CCR2*), and further validated the role of *CASP1* in AS through both in vitro and in vivo experiments. These findings may provide new insights into the diagnosis and treatment of atherosclerosis.

## Supporting information

S1 Raw Images The original blots. (PDF)

S1 Table Types and Mechanisms of Action of 23 CASP1-Targeted Drugs. (CSV)

S1 Fig Comparative ROC curve plots of the five core genes. (EMF)

## References

[1] Tamargo IA, Baek KI, Kim Y, Park C, Jo H. Flow-induced reprogramming of endothelial cells in atherosclerosis. Nat Rev Cardiol. 2023;20(11):738–53. doi: 10.1038/s41569-023-00883-1 37225873 PMC10206587

[2] Soehnlein O, Libby P. Targeting inflammation in atherosclerosis - from experimental insights to the clinic. Nat Rev Drug Discov. 2021;20(8):589–610. doi: 10.1038/s41573-021-00198-1 33976384 PMC8112476

[3] Hao X-T, Peng R, Guan M, Zhang H-J, Guo Y, Shalapy NM, et al. The food and medicinal homological resources benefiting patients with hyperlipidemia: categories, functional components, and mechanisms. Food & Medicine Homology. 2024;1(2):9420003. doi: 10.26599/fmh.2024.9420003

[4] Fan J, Watanabe T. Atherosclerosis: Known and unknown. Pathol Int. 2022;72(3):151–60. doi: 10.1111/pin.13202 35076127

[5] Gallego-Colon E, Daum A, Yosefy C. Statins and PCSK9 inhibitors: a new lipid-lowering therapy. Eur J Pharmacol. 2020;878:173114. doi: 10.1016/j.ejphar.2020.173114 32302598

[6] Yamamoto H, Zhang S, Mizushima N. Autophagy genes in biology and disease. Nat Rev Genet. 2023;24(6):382–400. doi: 10.1038/s41576-022-00562-w 36635405 PMC9838376

[7] Wang Z, Sequeira RC, Zabalawi M, Madenspacher J, Boudyguina E, Ou T, et al. Myeloid atg5 deletion impairs n-3 PUFA-mediated atheroprotection. Atherosclerosis. 2020;295:8–17. doi: 10.1016/j.atherosclerosis.2020.01.004 31978760 PMC7034781

[8] Madrigal-Matute J, de Bruijn J, van Kuijk K, Riascos-Bernal DF, Diaz A, Tasset I, et al. Protective role of chaperone-mediated autophagy against atherosclerosis. Proc Natl Acad Sci U S A. 2022;119(14):e2121133119. doi: 10.1073/pnas.2121133119 35363568 PMC9168839

[9] Zhang Y, Vandestienne M, Lavillegrand J-R, Joffre J, Santos-Zas I, Lavelle A, et al. Genetic inhibition of CARD9 accelerates the development of atherosclerosis in mice through CD36 dependent-defective autophagy. Nat Commun. 2023;14(1):4622. doi: 10.1038/s41467-023-40216-x 37528097 PMC10394049

[10] Liang M, Wang Q, Zhang S, Lan Q, Wang R, Tan E, et al. Polypyridiniums with inherent autophagy-inducing activity for atherosclerosis treatment by intracellularly co-delivering two antioxidant enzymes. Adv Mater. 2024;36(46):e2409015. doi: 10.1002/adma.202409015 39328054

[11] Lin L, Zhang MX, Zhang L, Zhang D, Li C, Li YL. Autophagy, pyroptosis, and ferroptosis: new regulatory mechanisms for atherosclerosis. Frontiers in Cell and Developmental Biology. 2021;9:809955. doi: 10.3389/fcell.2021.809955 35096837 PMC8793783

[12] Qiao L, Ma J, Zhang Z, Sui W, Zhai C, Xu D, et al. Deficient chaperone-mediated autophagy promotes inflammation and atherosclerosis. Circ Res. 2021;129(12):1141–57. doi: 10.1161/CIRCRESAHA.121.318908 34704457 PMC8638823

[13] Zhu B-R, Li J, zhang Q. Refining the gut-microbiome axis: a triad of metabolites, targeted microbial delivery, and AI-assisted profiling for precision medicine-food intervention. Food & Medicine Homology. 2025;2(4):9420118. doi: 10.26599/fmh.2025.9420118

[14] Zhou G, Soufan O, Ewald J, Hancock REW, Basu N, Xia J. NetworkAnalyst 3.0: a visual analytics platform for comprehensive gene expression profiling and meta-analysis. Nucleic Acids Res. 2019;47(W1):W234–41. doi: 10.1093/nar/gkz240 30931480 PMC6602507

[15] Cannon M, Stevenson J, Stahl K, Basu R, Coffman A, Kiwala S, et al. DGIdb 5.0: rebuilding the drug-gene interaction database for precision medicine and drug discovery platforms. Nucleic Acids Res. 2024;52(D1):D1227–35. doi: 10.1093/nar/gkad1040 37953380 PMC10767982

[16] Roth GA, Mensah GA, Johnson CO, Addolorato G, Ammirati E, Baddour LM, et al. Global burden of cardiovascular diseases and risk factors, 1990-2019: update from the GBD 2019 study. J Am Coll Cardiol. 2020;76(25):2982–3021. doi: 10.1016/j.jacc.2020.11.010 33309175 PMC7755038

[17] Ciumărnean L, Milaciu MV, Negrean V, Orășan OH, Vesa SC, Sălăgean O, et al. Cardiovascular risk factors and physical activity for the prevention of cardiovascular diseases in the elderly. Int J Environ Res Public Health. 2021;19(1):207. doi: 10.3390/ijerph19010207 35010467 PMC8751147

[18] Parikh NS, Gottesman RF. Midlife cardiovascular risk factors, subclinical atherosclerosis, and cerebral hypometabolism. J Am Coll Cardiol. 2021;77(7):899–901. doi: 10.1016/j.jacc.2020.12.046 33602473

[19] Zhang T-T, Cai Q-L, Gu Z-Y, Song T-Y. Novel ACE-inhibiting peptides from soybean protein hydrolysates by peptidomics combined with in silico analysis and their inhibitory effects on proliferation and migration of Ang II-induced VSMCs. Food & Medicine Homology. 2024;1(2):9420023. doi: 10.26599/fmh.2024.9420023

[20] Zhou H-Q, Zhang L-M, Li X, Huang Z-H. Crosstalk between autophagy and inflammation in chronic cerebral ischaemia. Cell Mol Neurobiol. 2023;43(6):2557–66. doi: 10.1007/s10571-023-01336-6 36952071 PMC10333405

[21] Engelen SE, Robinson AJB, Zurke Y-X, Monaco C. Therapeutic strategies targeting inflammation and immunity in atherosclerosis: how to proceed?. Nat Rev Cardiol. 2022;19(8):522–42. doi: 10.1038/s41569-021-00668-4 35102320 PMC8802279

[22] Li S, Huang Q, Zhou D, He B. PRKCD as a potential therapeutic target for chronic obstructive pulmonary disease. Int Immunopharmacol. 2022;113(Pt A):109374. doi: 10.1016/j.intimp.2022.109374 36279664

[23] Lien C-F, Chen S-J, Tsai M-C, Lin C-S. Potential role of protein kinase c in the pathophysiology of diabetes-associated atherosclerosis. Front Pharmacol. 2021;12:716332. doi: 10.3389/fphar.2021.716332 34276388 PMC8283198

[24] Leitges M, Mayr M, Braun U, Mayr U, Li C, Pfister G, et al. Exacerbated vein graft arteriosclerosis in protein kinase Cdelta-null mice. J Clin Invest. 2001;108(10):1505–12. doi: 10.1172/JCI12902 11714742 PMC209416

[25] Qin P, He C, Ye P, Li Q, Cai C, Li Y. PKCδ regulates the vascular biology in diabetic atherosclerosis. Cell Commun Signal. 2023;21(1):330. doi: 10.1186/s12964-023-01361-4 37974282 PMC10652453

[26] Kesavardhana S, Malireddi RKS, Kanneganti T-D. Caspases in cell death, inflammation, and pyroptosis. Annu Rev Immunol. 2020;38:567–95. doi: 10.1146/annurev-immunol-073119-095439 32017655 PMC7190443

[27] Exconde PM, Hernandez-Chavez C, Bourne CM, Richards RM, Bray MB, Lopez JL, et al. The tetrapeptide sequence of IL-18 and IL-1β regulates their recruitment and activation by inflammatory caspases. Cell Rep. 2023;42(12):113581. doi: 10.1016/j.celrep.2023.113581 38103201 PMC11158830

[28] Huang P, Yan L, Li Z, Zhao S, Feng Y, Zeng J, et al. Potential shared gene signatures and molecular mechanisms between atherosclerosis and depression: evidence from transcriptome data. Comput Biol Med. 2023;152:106450. doi: 10.1016/j.compbiomed.2022.106450 36565484

[29] Kirii H, Niwa T, Yamada Y, Wada H, Saito K, Iwakura Y, et al. Lack of interleukin-1beta decreases the severity of atherosclerosis in ApoE-deficient mice. Arterioscler Thromb Vasc Biol. 2003;23(4):656–60. doi: 10.1161/01.ATV.0000064374.15232.C3 12615675

[30] Bahrami A, Sathyapalan T, Sahebkar A. The role of interleukin-18 in the development and progression of atherosclerosis. Curr Med Chem. 2021;28(9):1757–74. doi: 10.2174/0929867327666200427095830 32338205

[31] O’Brien ME, Murray G, Gogoi D, Yusuf A, McCarthy C, Wormald MR, et al. A review of alpha-1 antitrypsin binding partners for immune regulation and potential therapeutic application. Int J Mol Sci. 2022;23(5):2441. doi: 10.3390/ijms23052441 35269582 PMC8910375

[32] Mahta A, Yaghi S, Reznik ME, Thompson BB, Wendell LC, Rao S, et al. Serum alpha-1 antitrypsin in acute ischemic stroke: a prospective pilot study. J Clin Neurosci. 2020;76:20–4. doi: 10.1016/j.jocn.2020.04.074 32327380

[33] Winther SV, Landt EM, Nordestgaard BG, Seersholm N, Dahl M. α1-Antitrypsin deficiency associated with increased risk of heart failure. ERJ Open Res. 2023;9(5):00319–2023. doi: 10.1183/23120541.00319-2023 37753284 PMC10518873

[34] Wang Z, Xia Q, Su W, Zhang M, Gu Y, Xu J, et al. The commonness in immune infiltration of rheumatoid arthritis and atherosclerosis: screening for central targets via microarray data analysis. Front Immunol. 2022;13:1013531. doi: 10.3389/fimmu.2022.1013531 36311761 PMC9606677

[35] Ma Q, Zhang N, You Y, Zhu J, Yu Z, Chen H, et al. CXCR4 blockade in macrophage promotes angiogenesis in ischemic hindlimb by modulating autophagy. J Mol Cell Cardiol. 2022;169:57–70. doi: 10.1016/j.yjmcc.2022.05.002 35597127

[36] Döring Y, Noels H, van der Vorst EPC, Neideck C, Egea V, Drechsler M, et al. Vascular CXCR4 limits atherosclerosis by maintaining arterial integrity: evidence from mouse and human studies. Circulation. 2017;136(4):388–403. doi: 10.1161/CIRCULATIONAHA.117.027646 28450349 PMC5777319

[37] Boring L, Gosling J, Cleary M, Charo IF. Decreased lesion formation in CCR2-/- mice reveals a role for chemokines in the initiation of atherosclerosis. Nature. 1998;394(6696):894–7. doi: 10.1038/29788 9732872

[38] Hou D, Pei C, Yu D, Yang G. miR-188-5p silencing improves cerebral ischemia/reperfusion injury by targeting Lin28a. Metab Brain Dis. 2023;38(7):2327–38. doi: 10.1007/s11011-023-01273-9 37572229

[39] MicroRNA miR-188-5p enhances SUMO2/3 conjugation by targeting SENP3 and alleviates focal cerebral ischemia/reperfusion injury in rats. Iran J Basic Med Sci. 2024;27(10):1260–7. doi: 10.22038/ijbms.2024.76165.16485 39229582 PMC11366937

