# Supplementary material for: CASP1 as a key autophagy-related gene in atherosclerosis: Identification by bioinformatics analysis and experimental validation
Source: PLoS One. 2026 Aug 19;21(8):e0353352. doi: 10.1371/journal.pone.0353352 (PMC13489422; doi:10.1371/journal.pone.0353352)

The catalogue number of the molecular weight marker：26616

Figure 10

1- eNOS

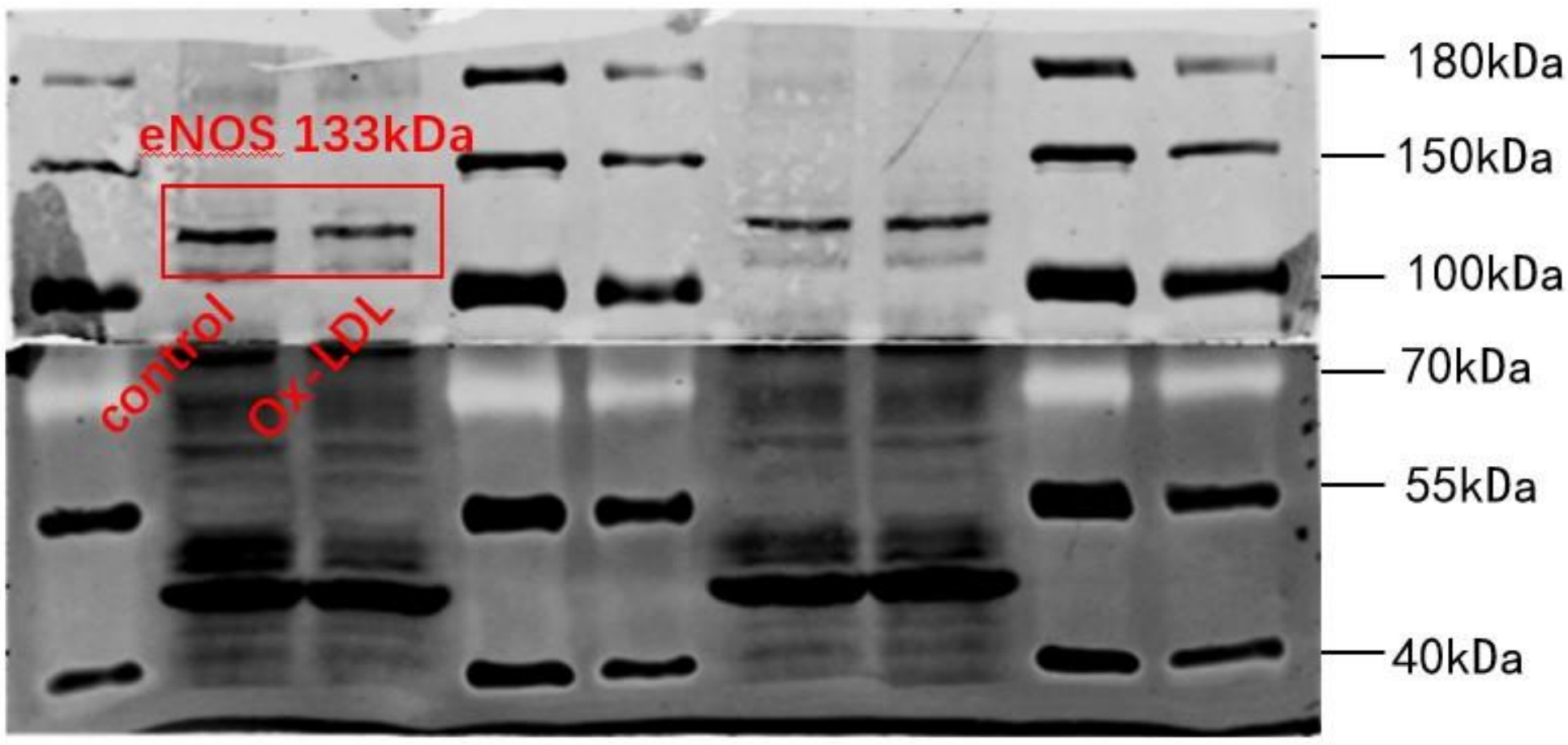

1-eNOS-actin

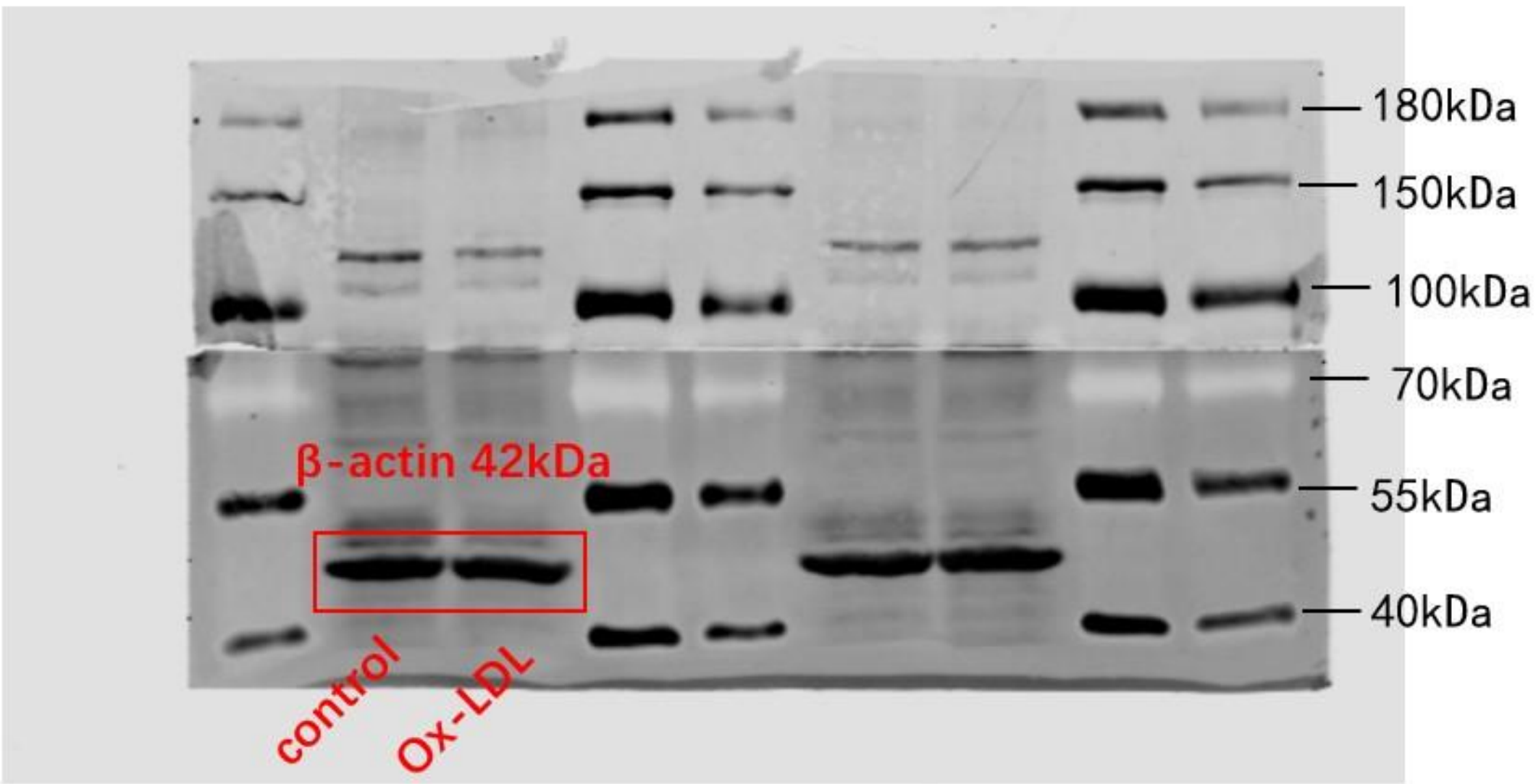

2-ICAM-1

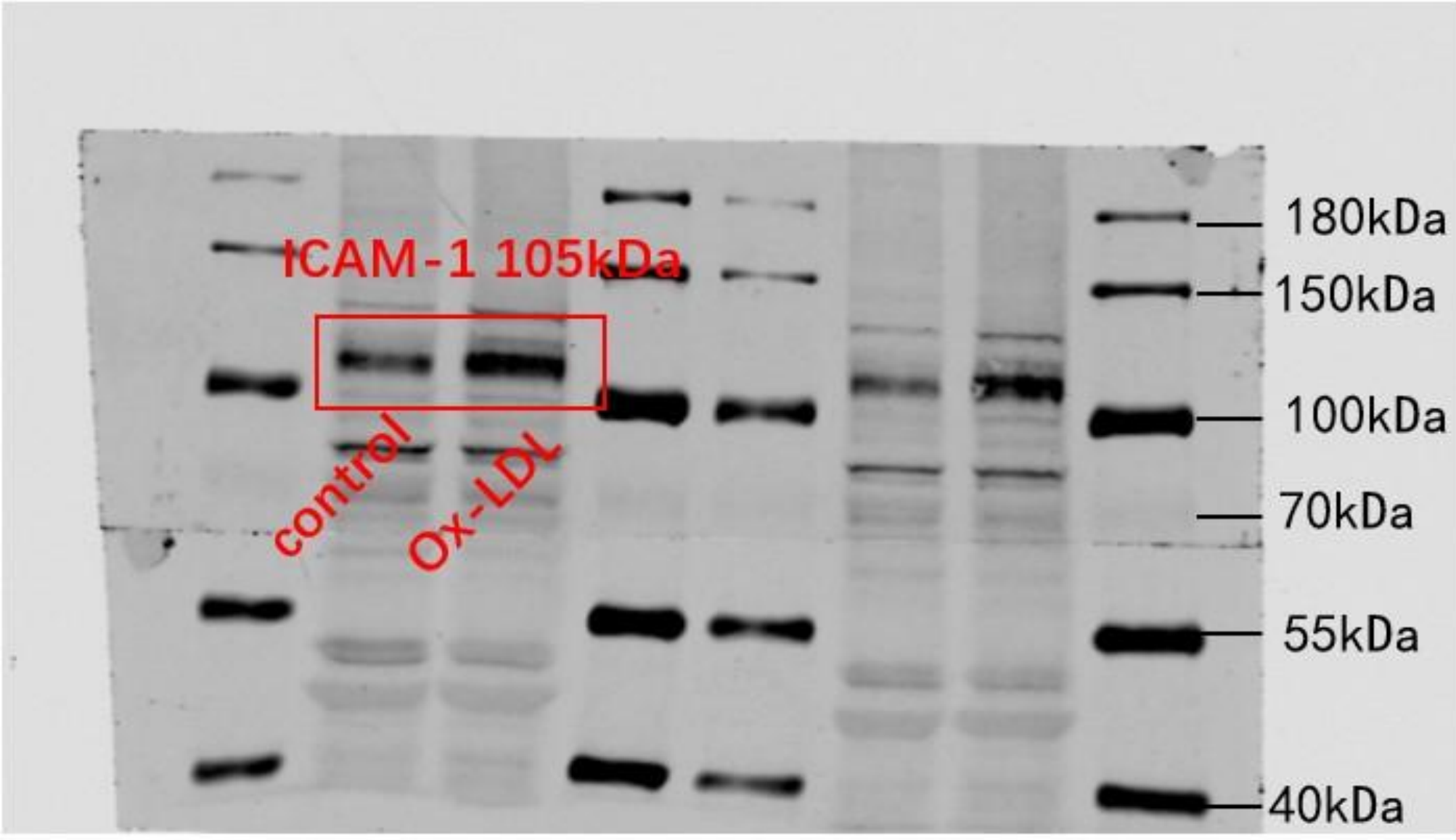

2-ICAM-1-actin

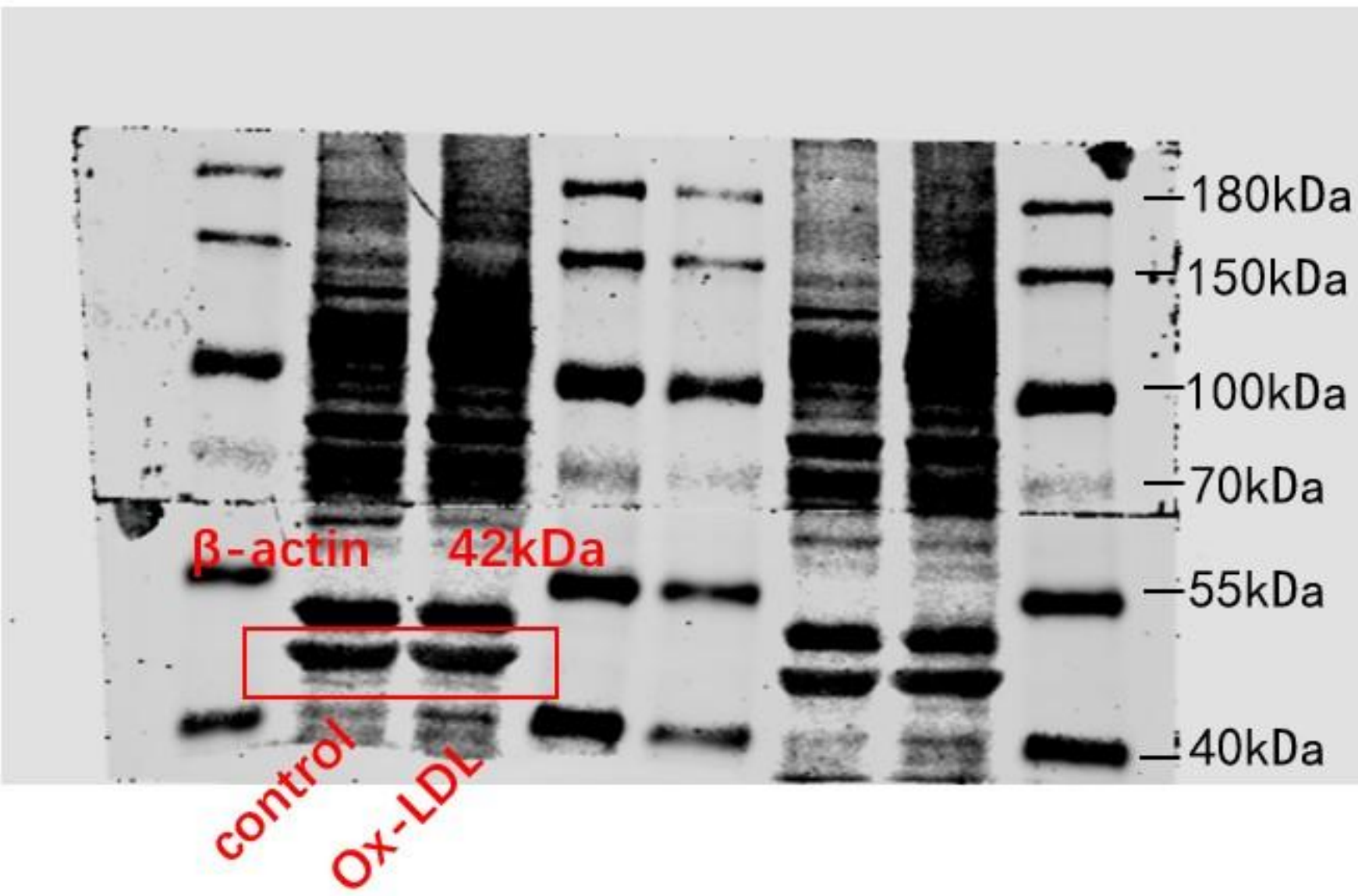

3-VCAM-1

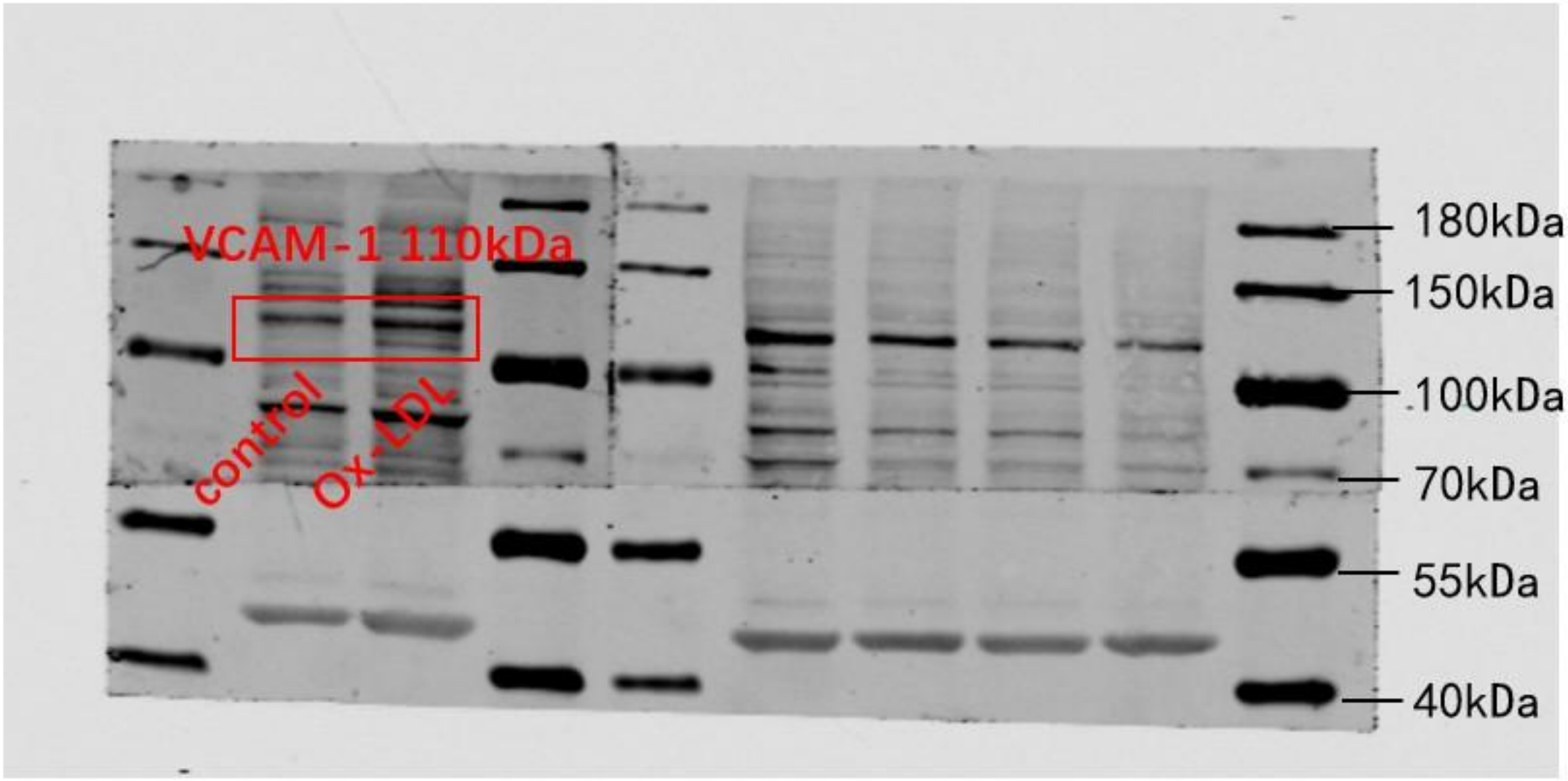

3-VCAM-1-actin

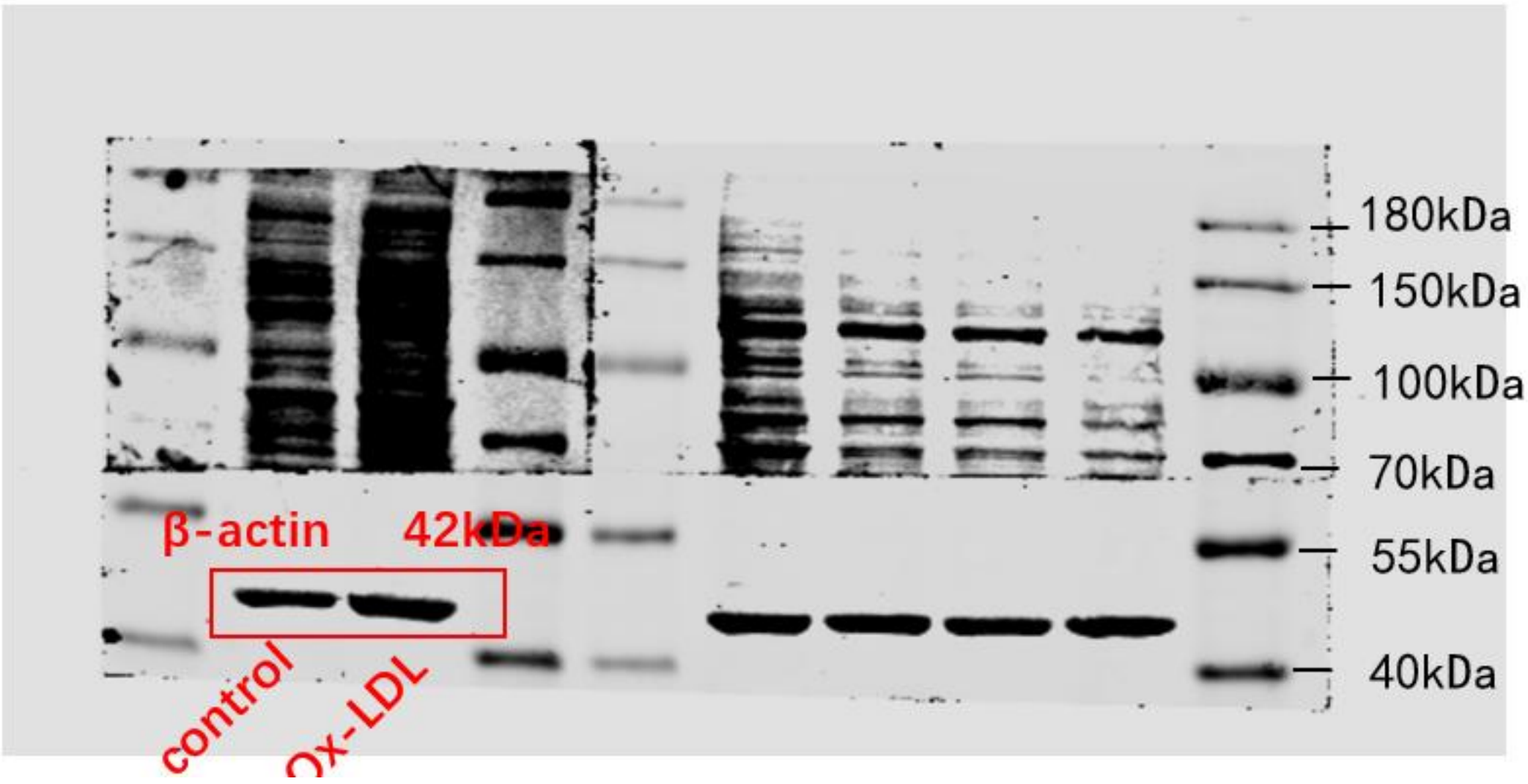

Figure 11

1-CASP1

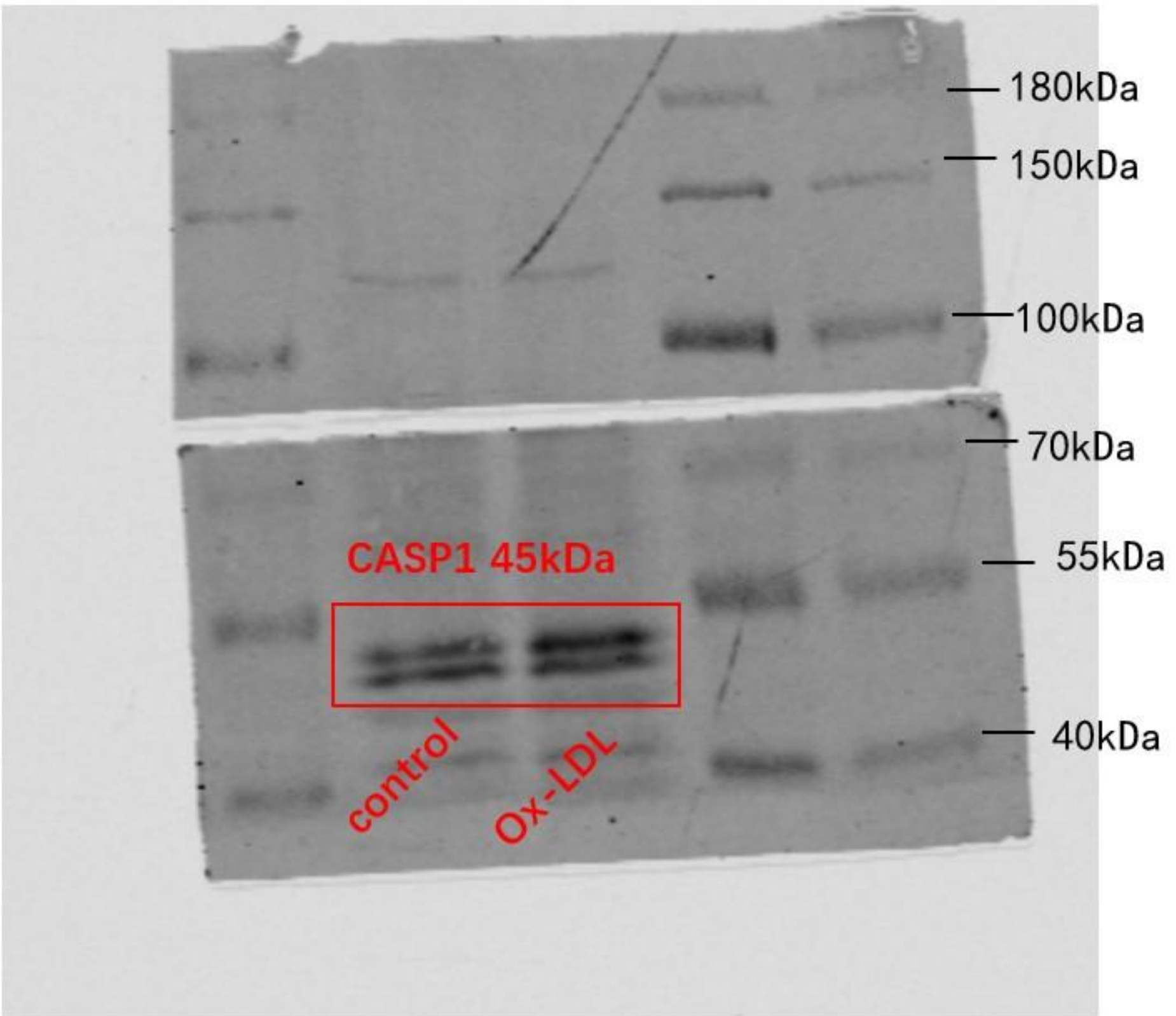

1--CASP1-β-actin

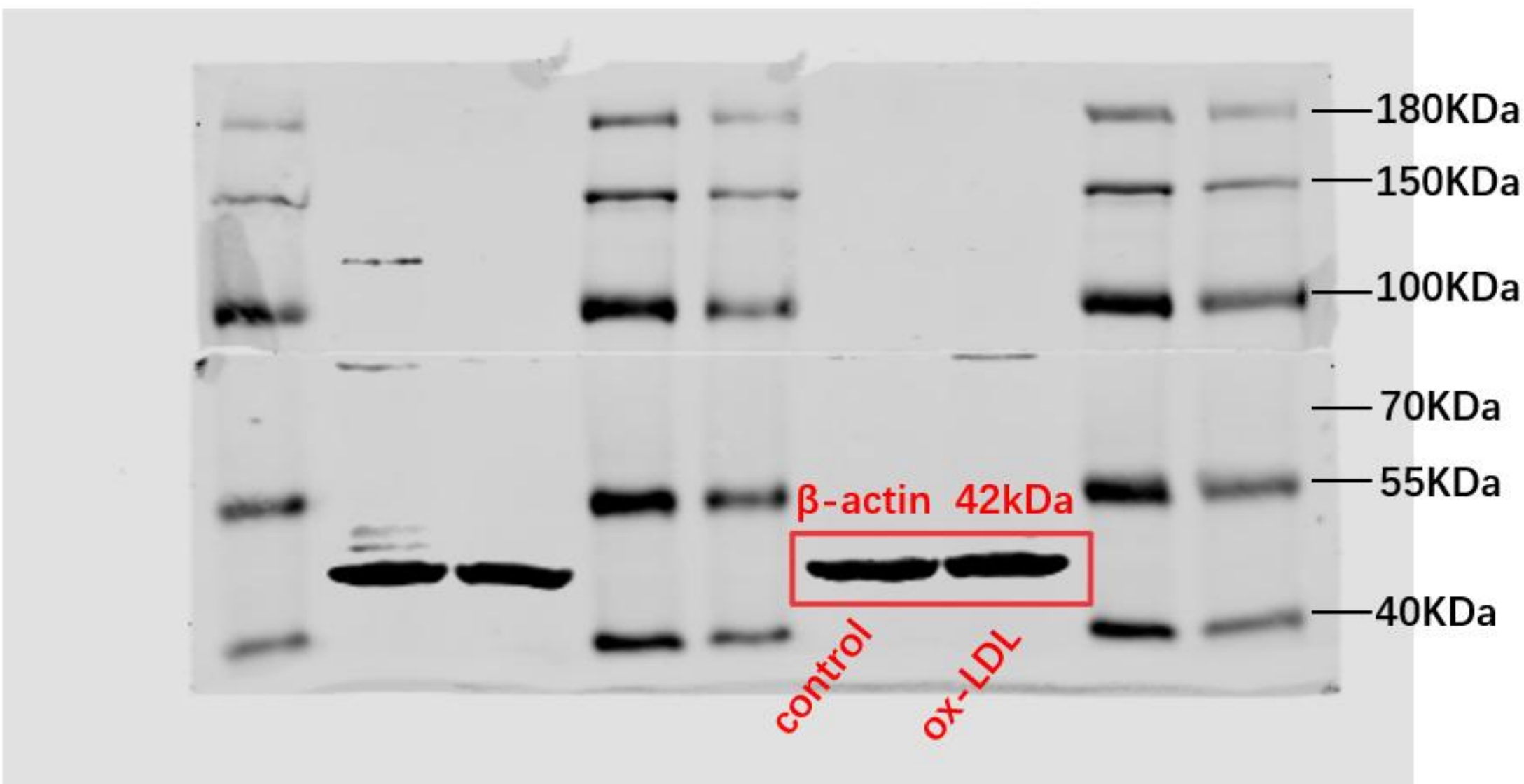

Figure 12

1-CASP1

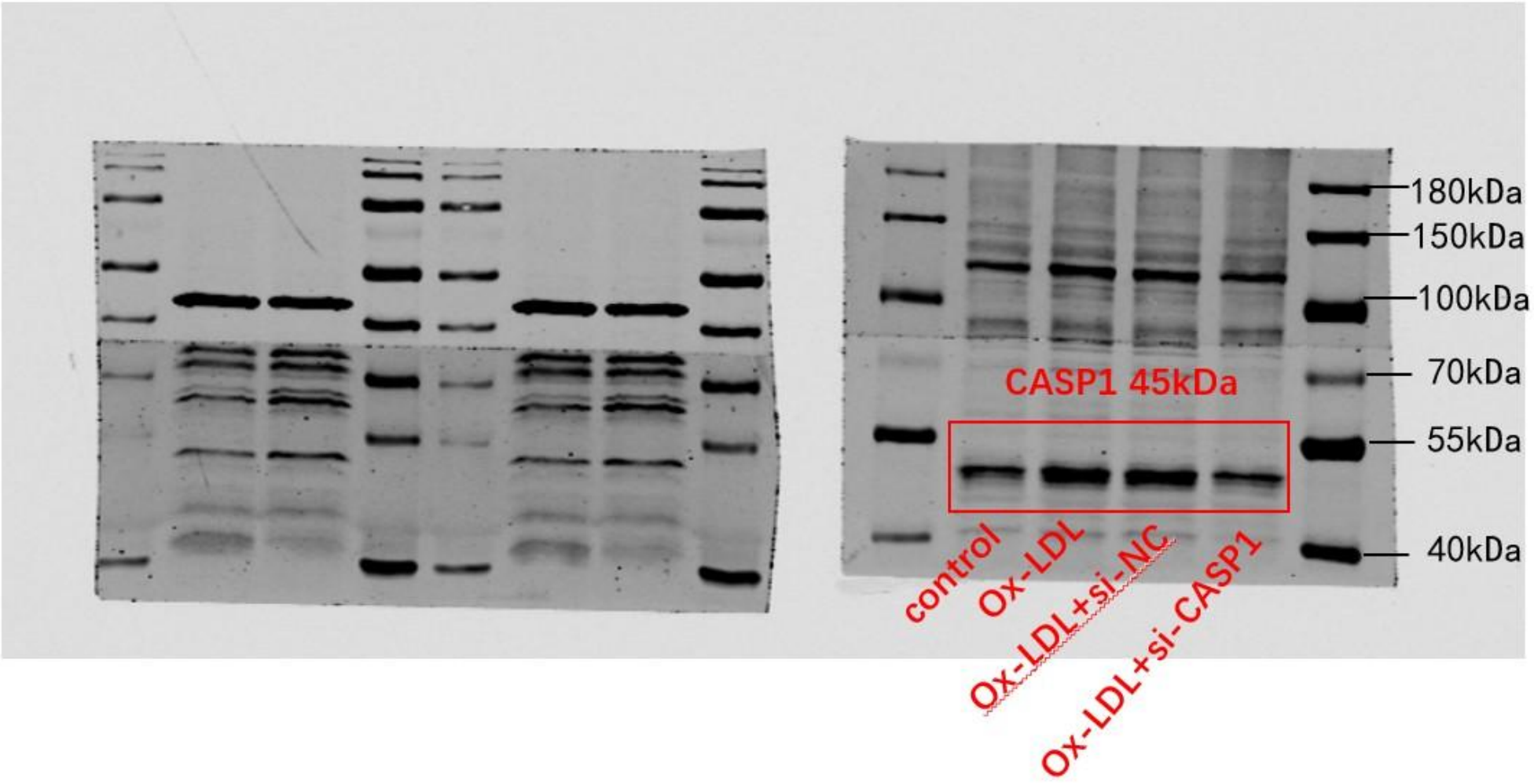

1-CASP1-actin

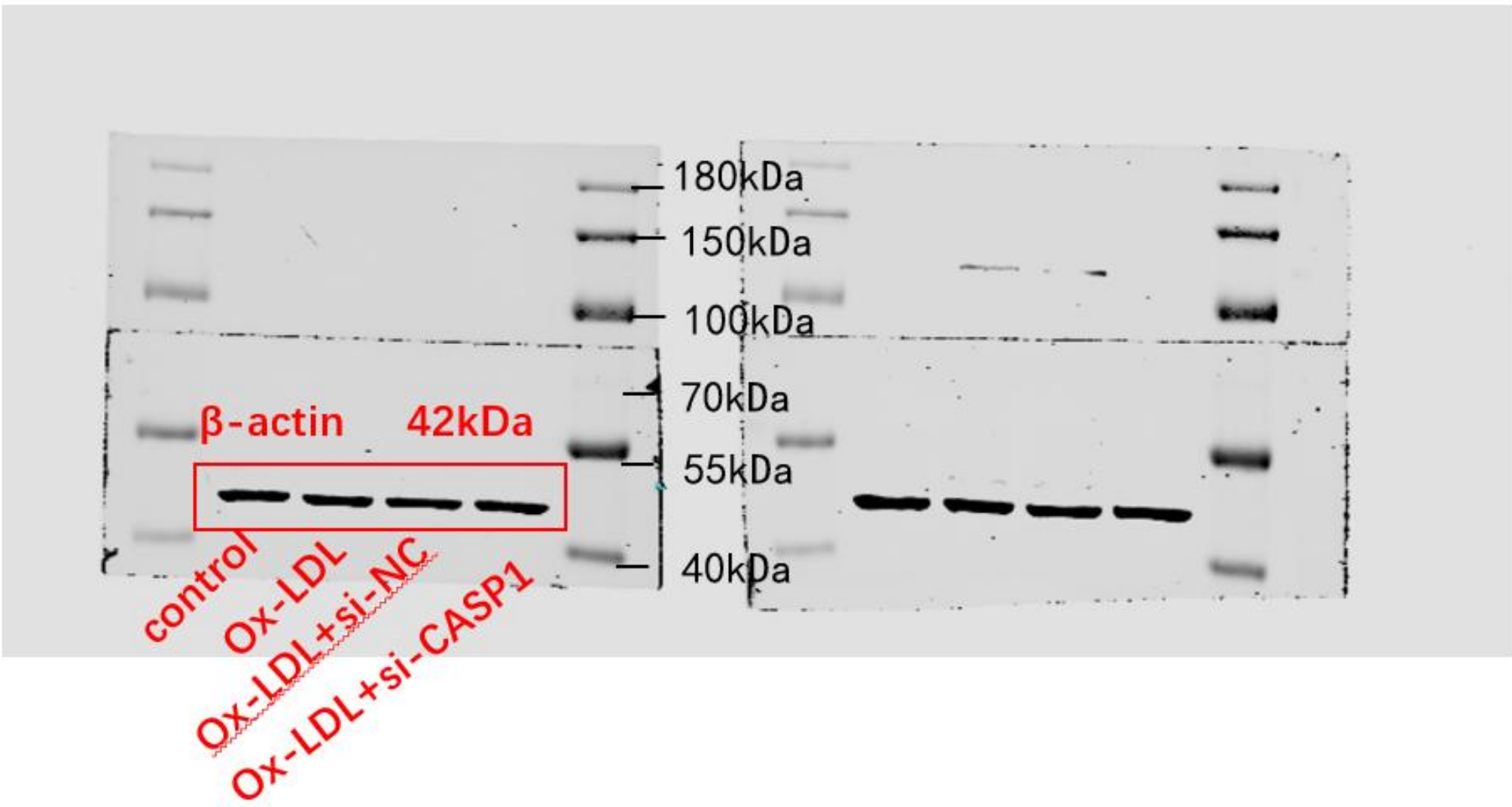

2-eNOS

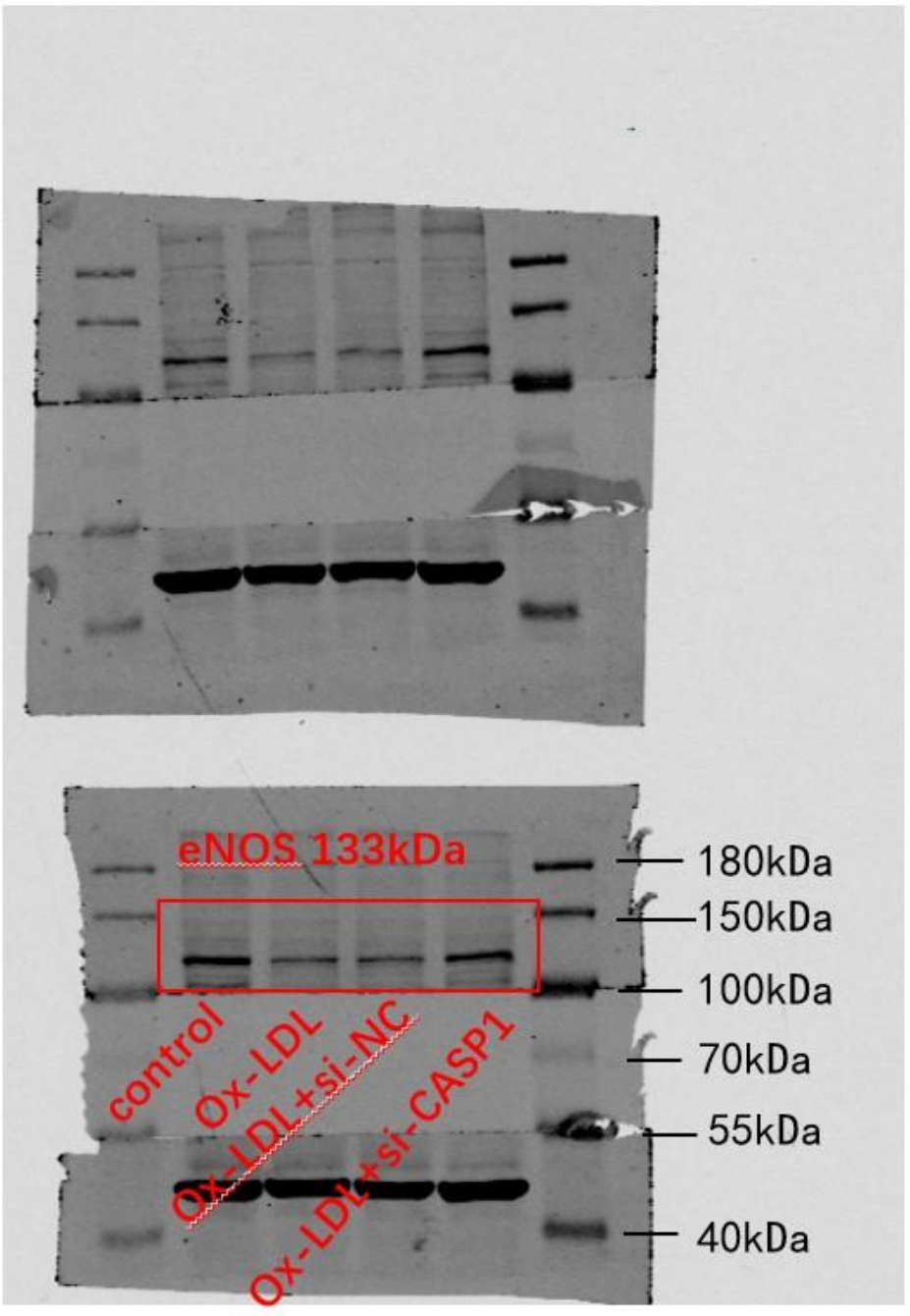

2-eNOS-actin

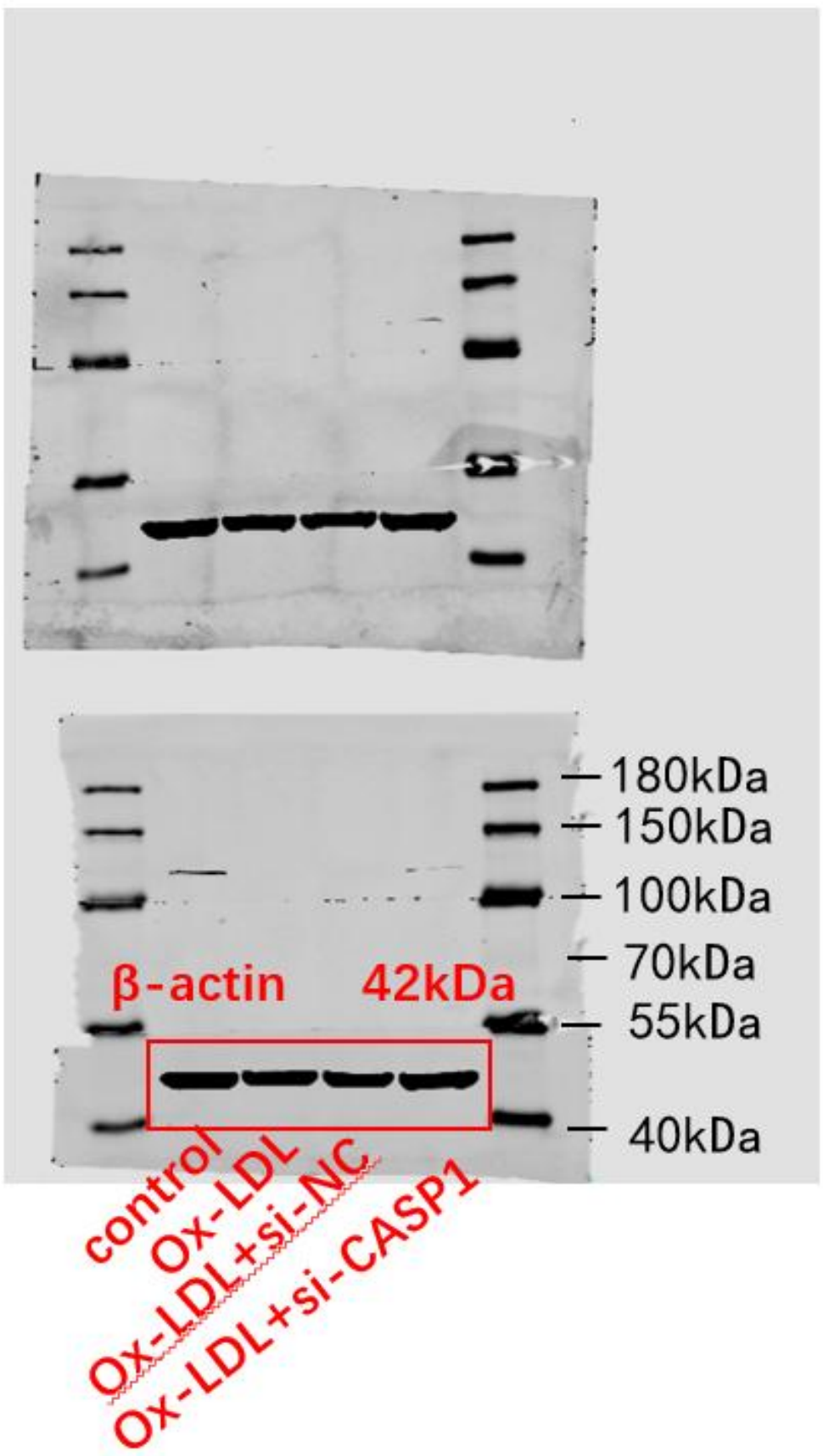

3-ICAM-1

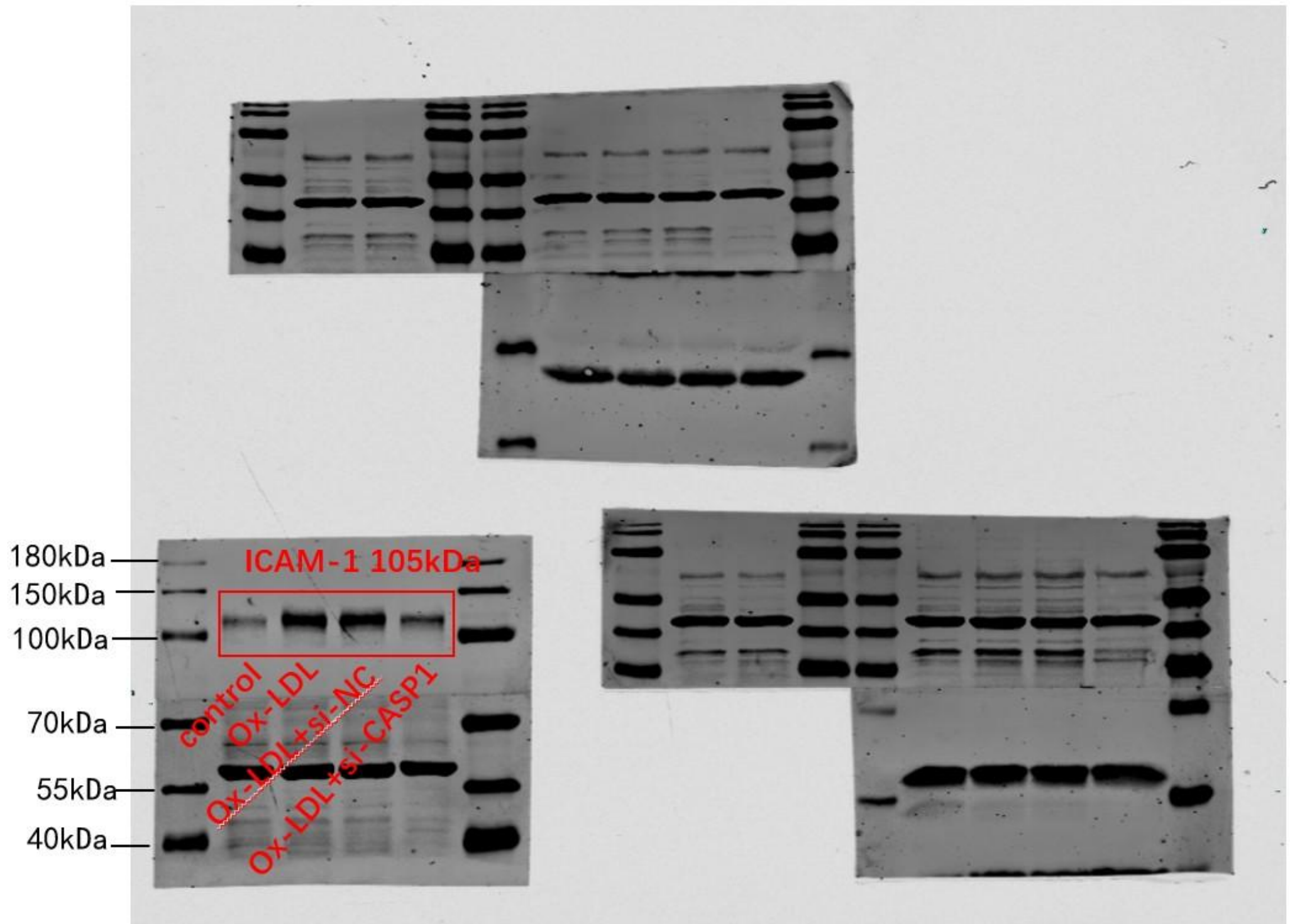

3-ICAM-1-actin

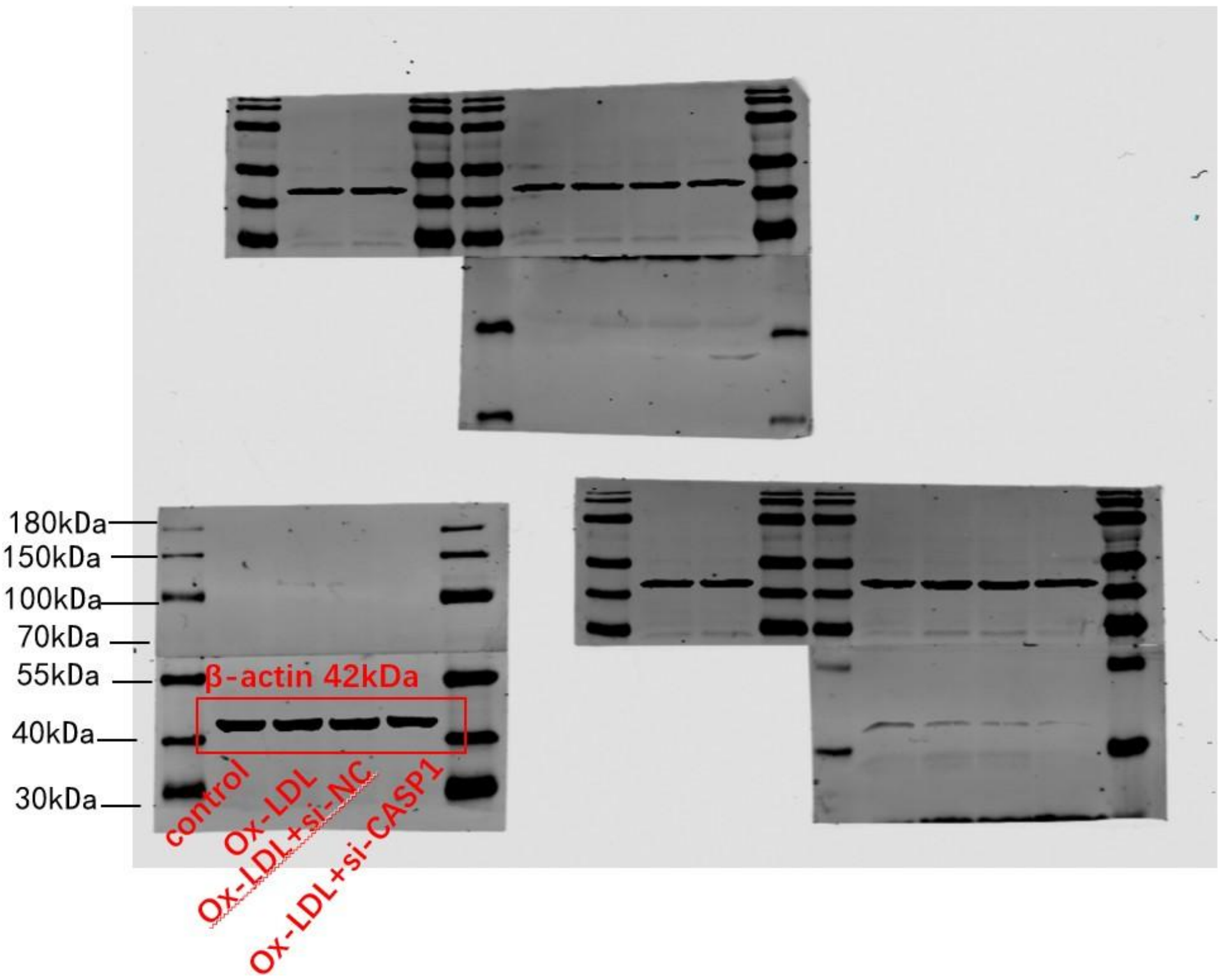

4-VCAM1

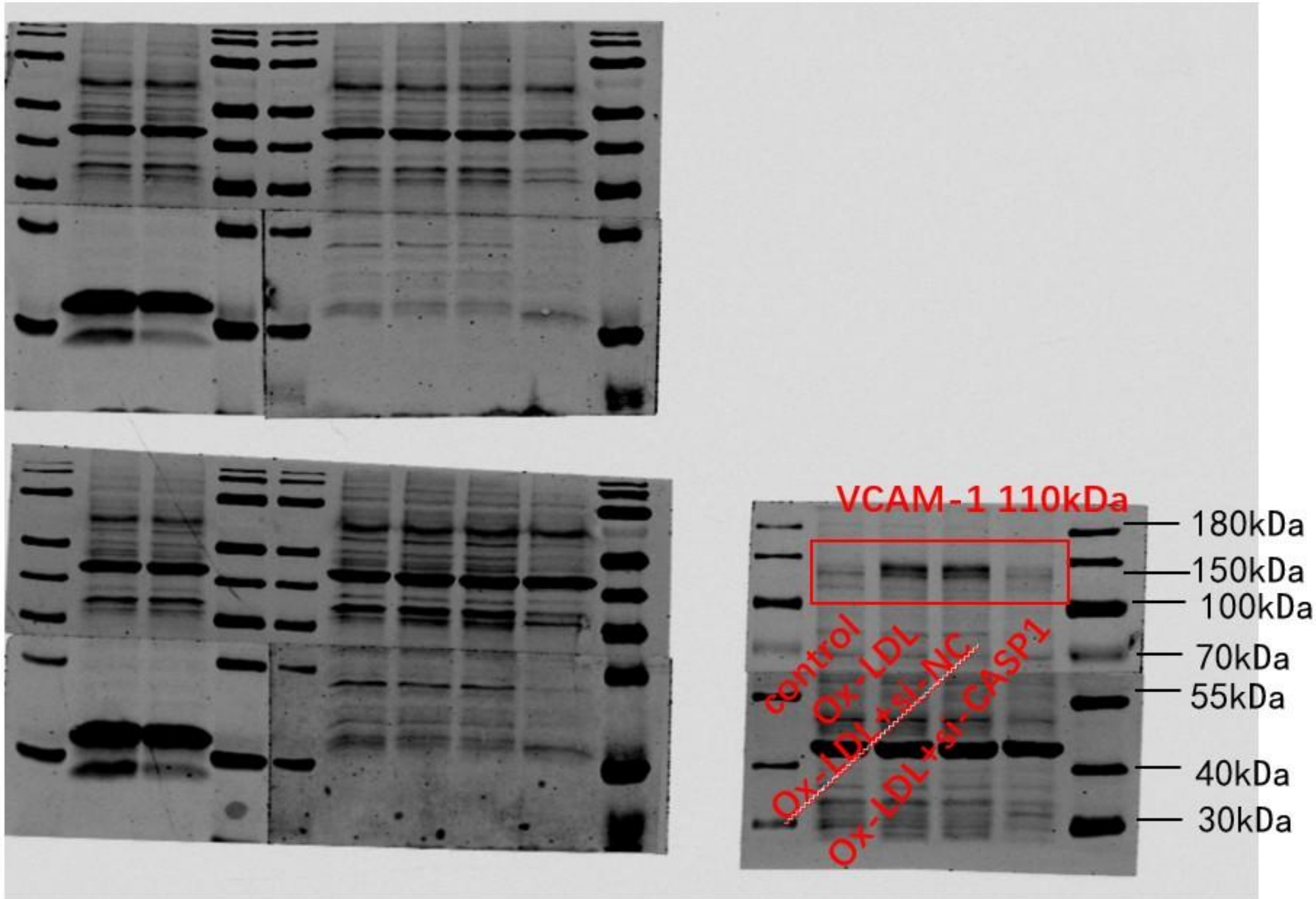

4-VCAM1-actin

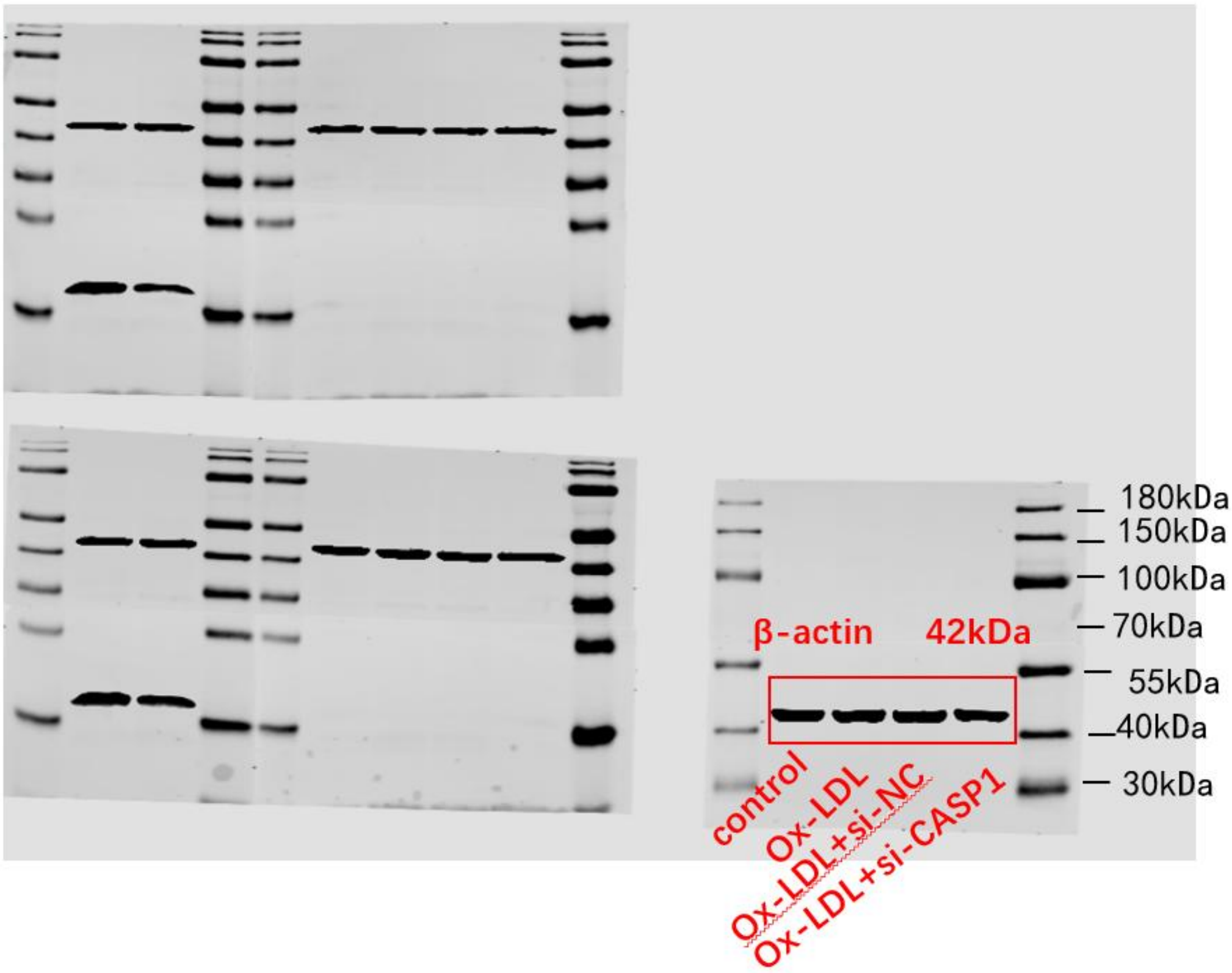

Figure 13

1- Beclin1

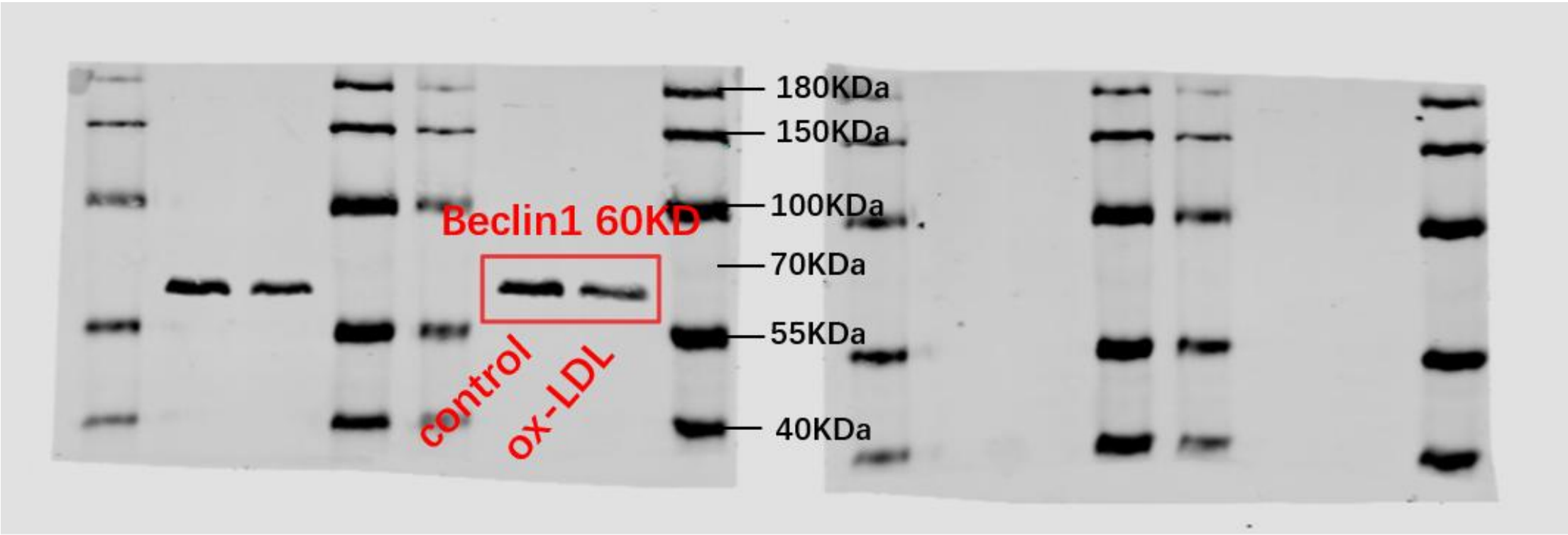

1- Beclin1-β-actin

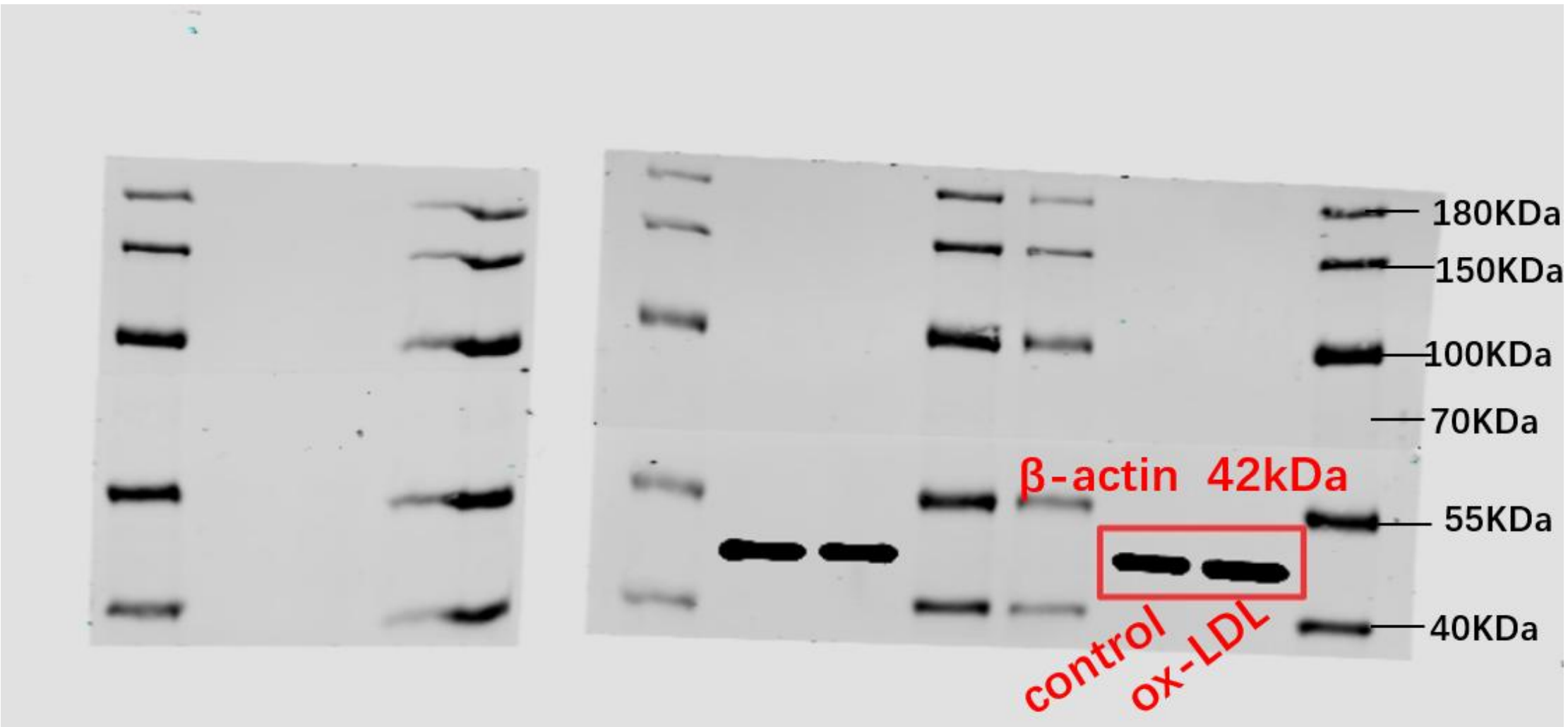

2-p62

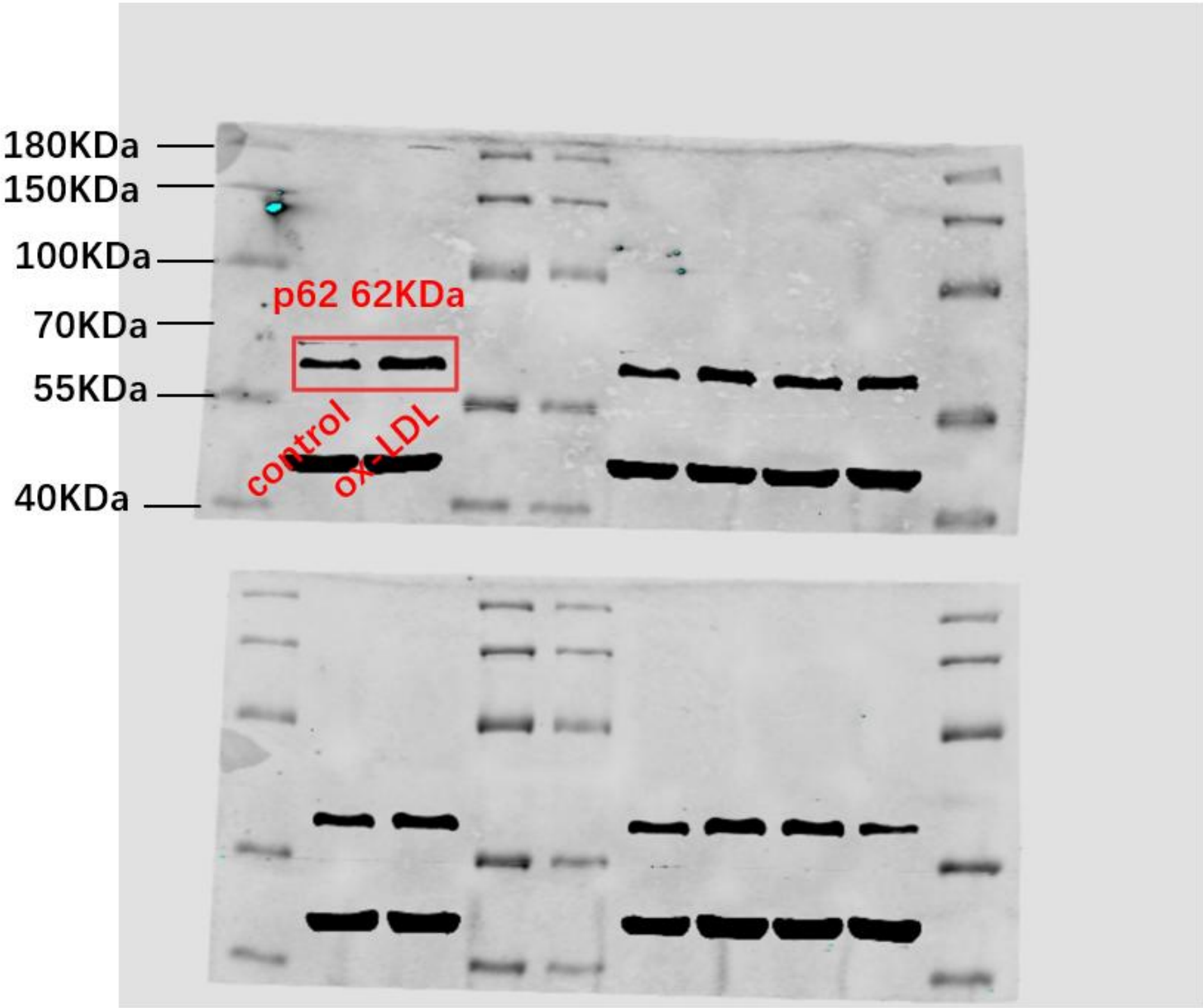

2- p62-β-actin

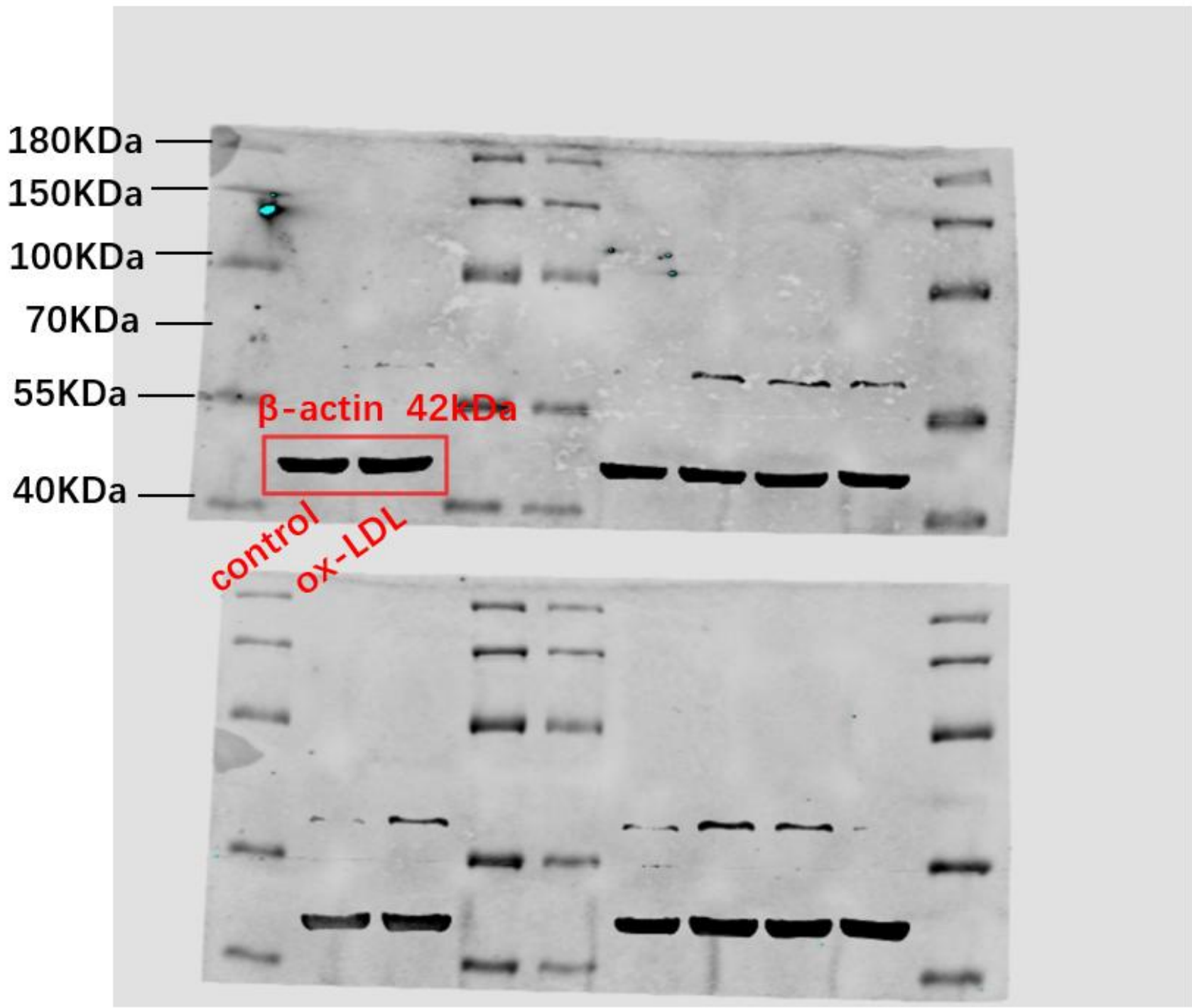

3-LC3II/LC3I

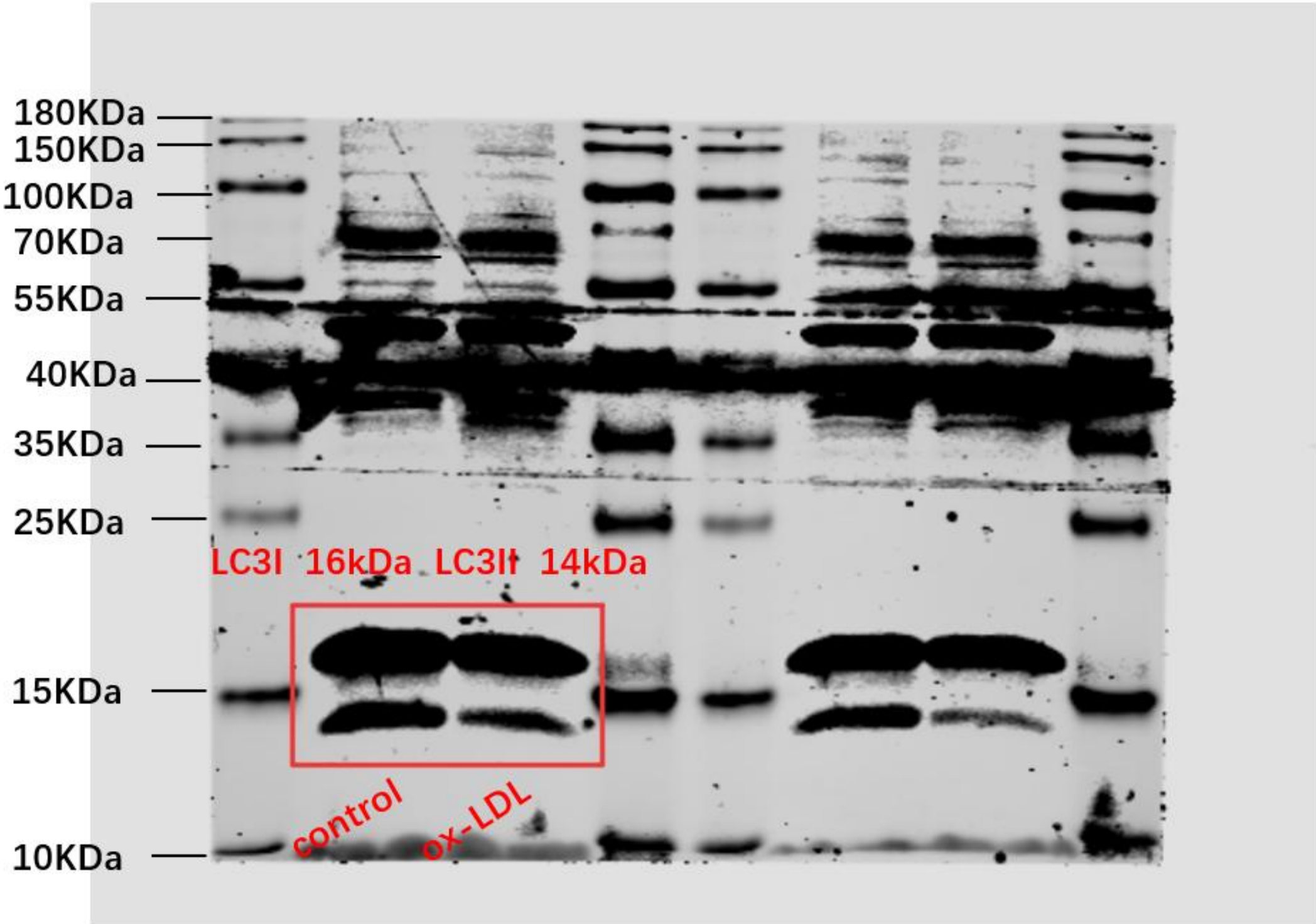

3-LC3II/LC3I-β-actin

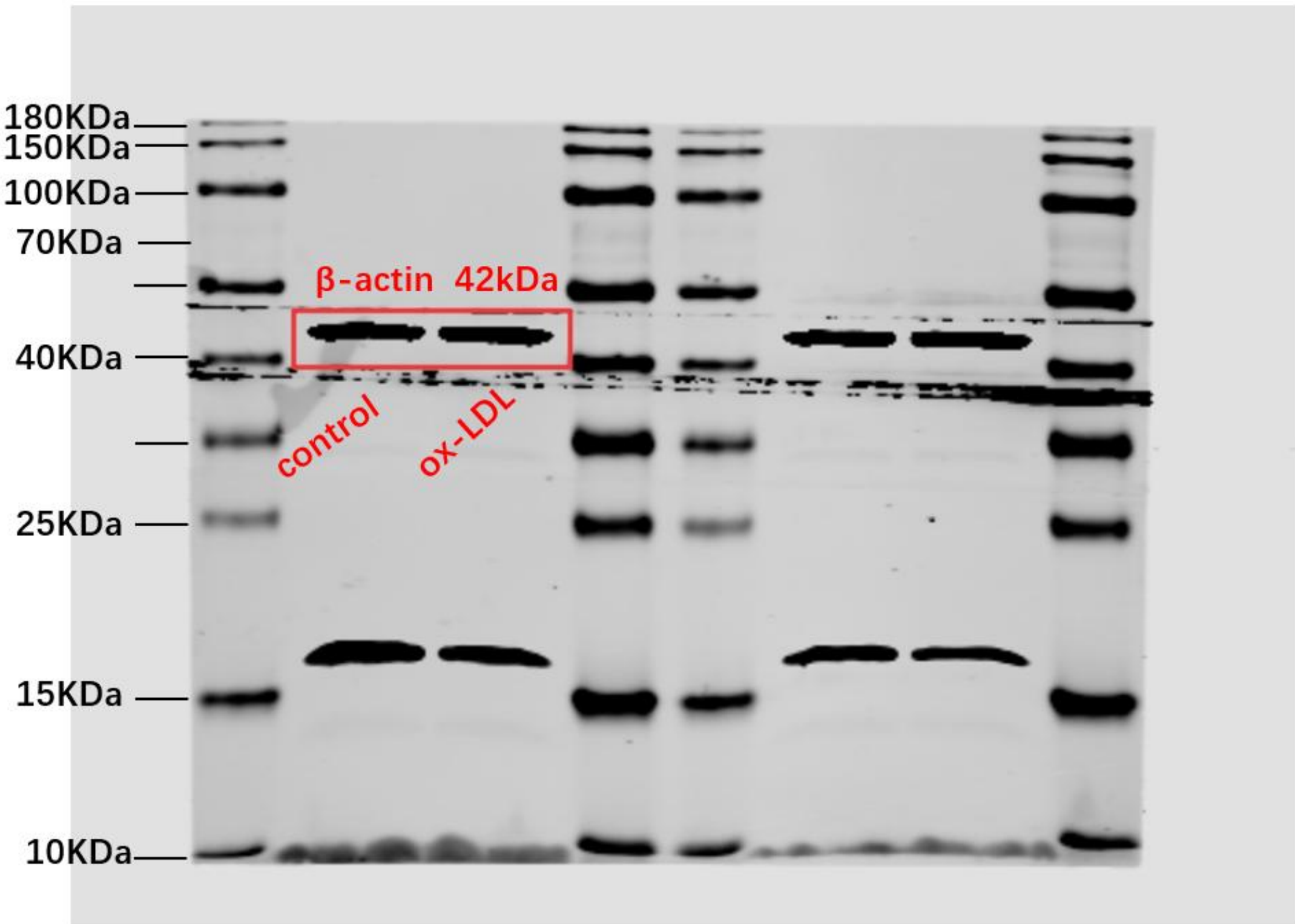

Figure 14

1- Beclin1

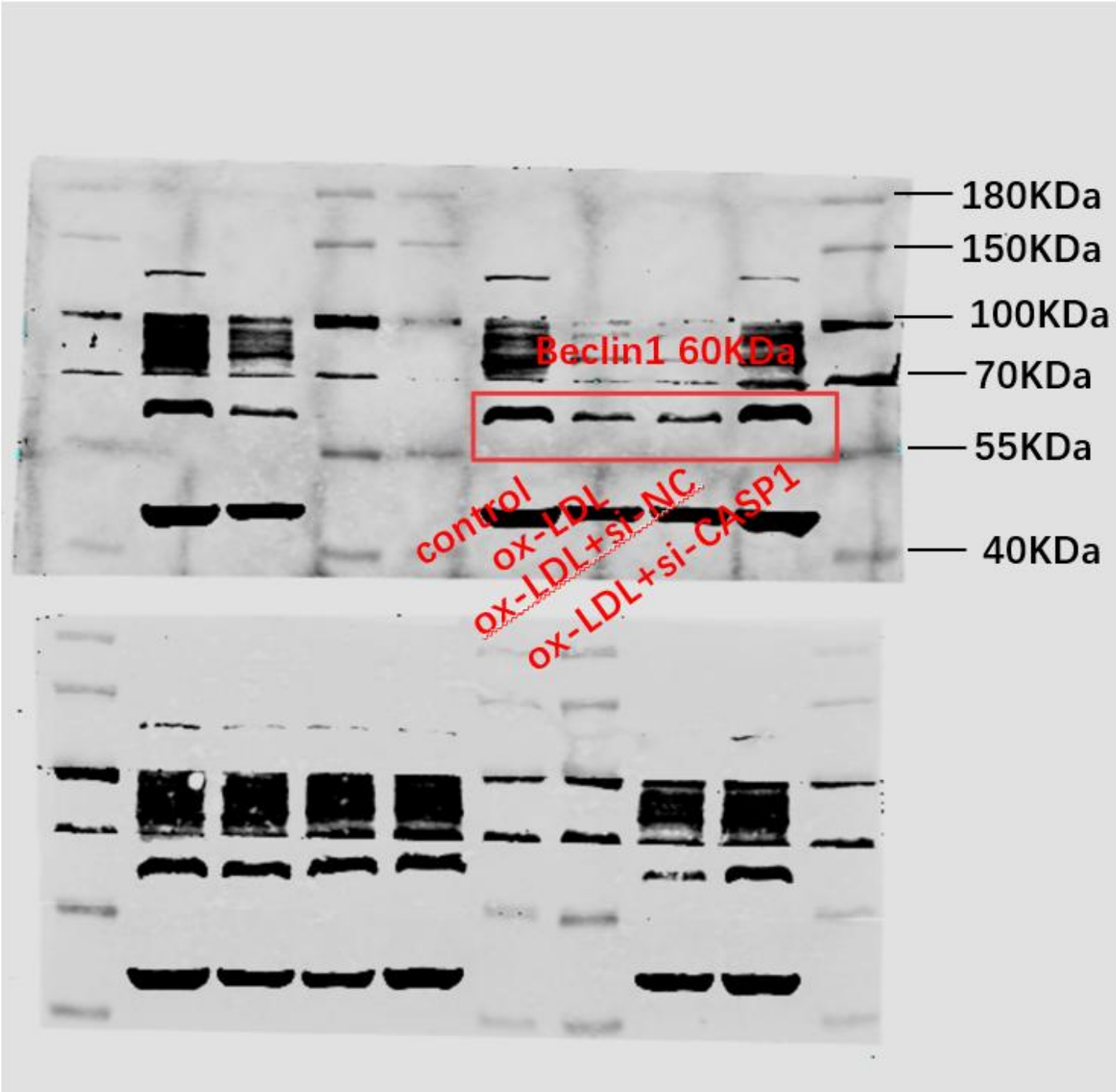

1- Beclin1-β-actin

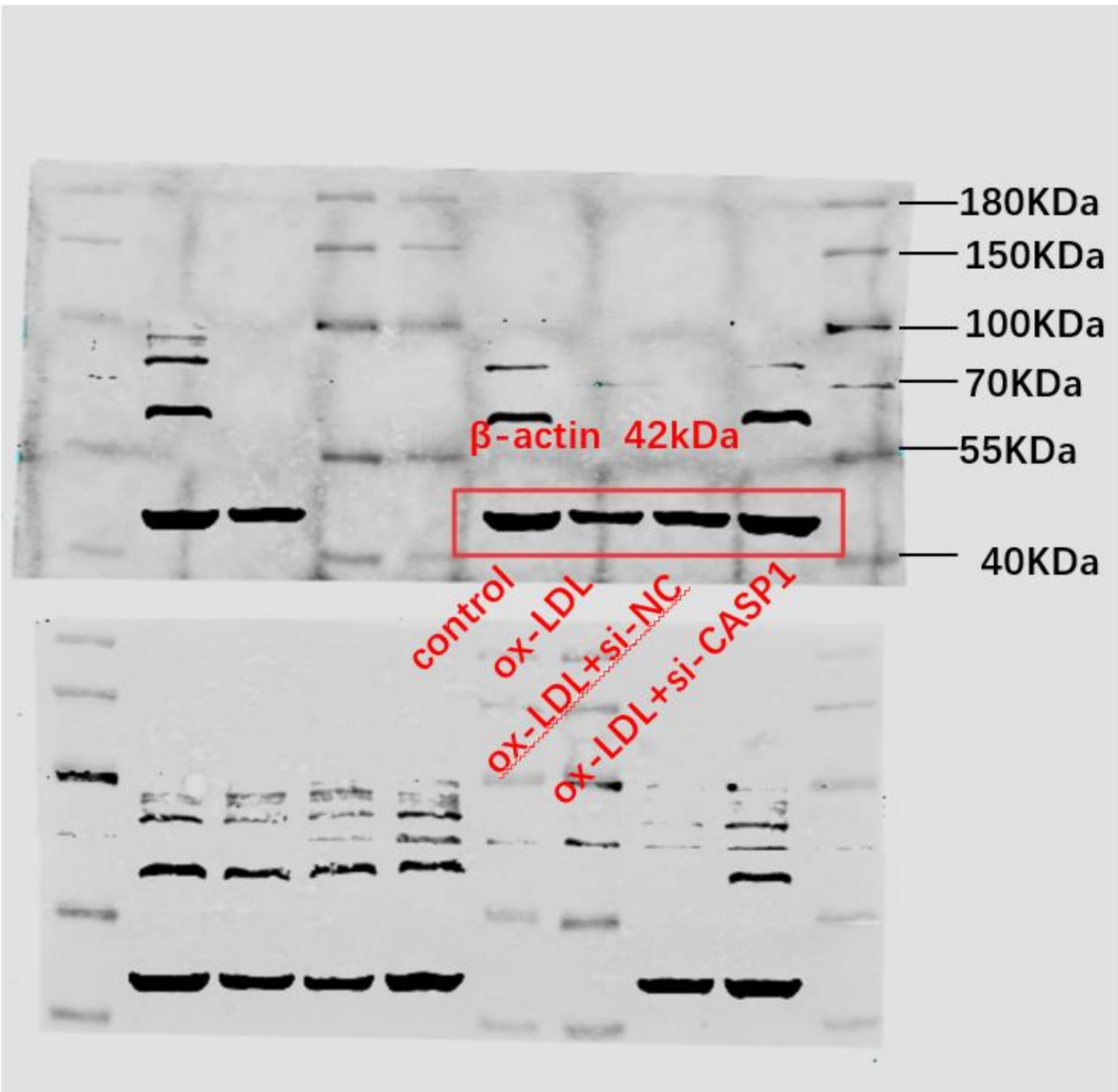

2-p62

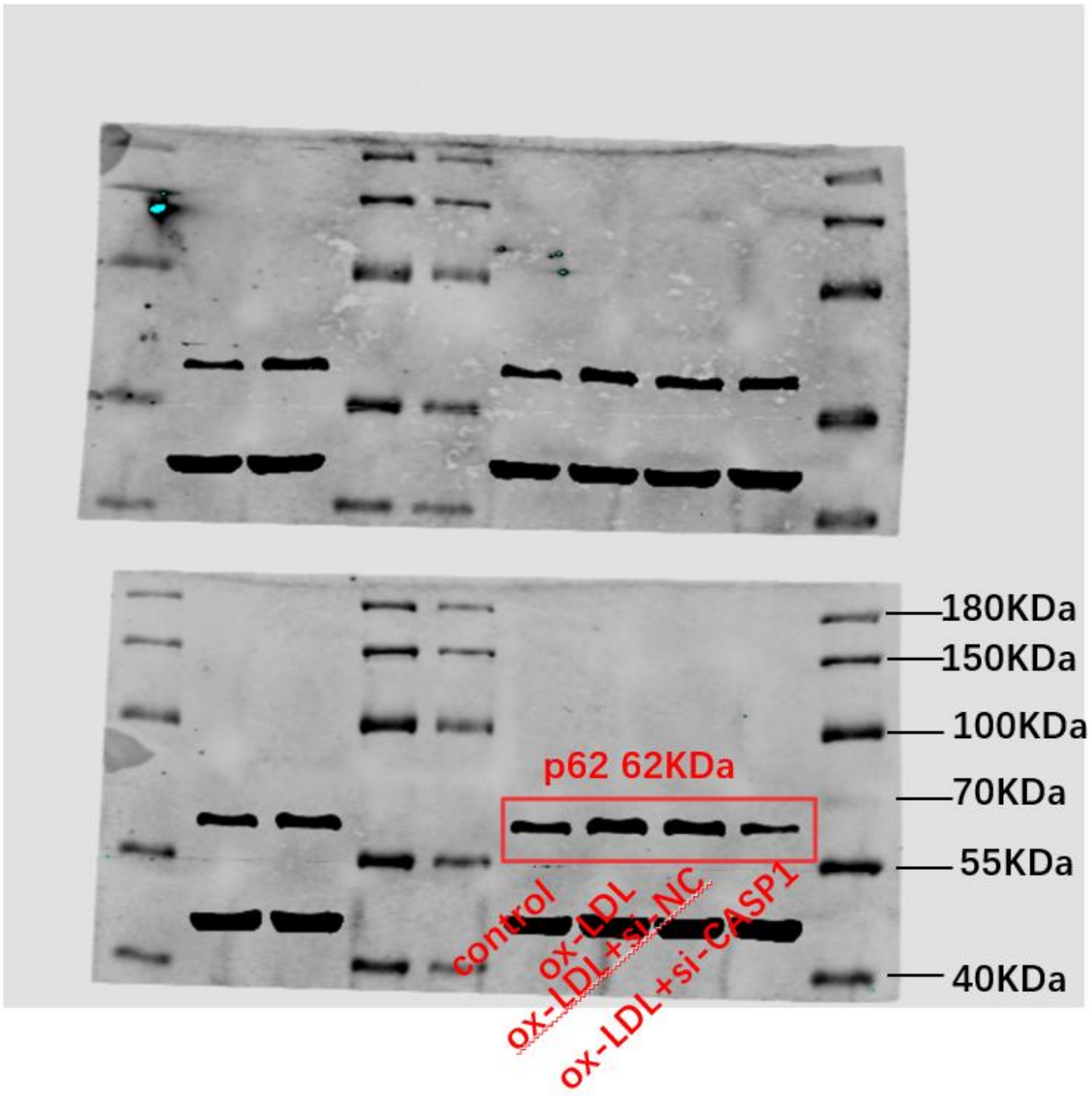

2- p62-β-actin

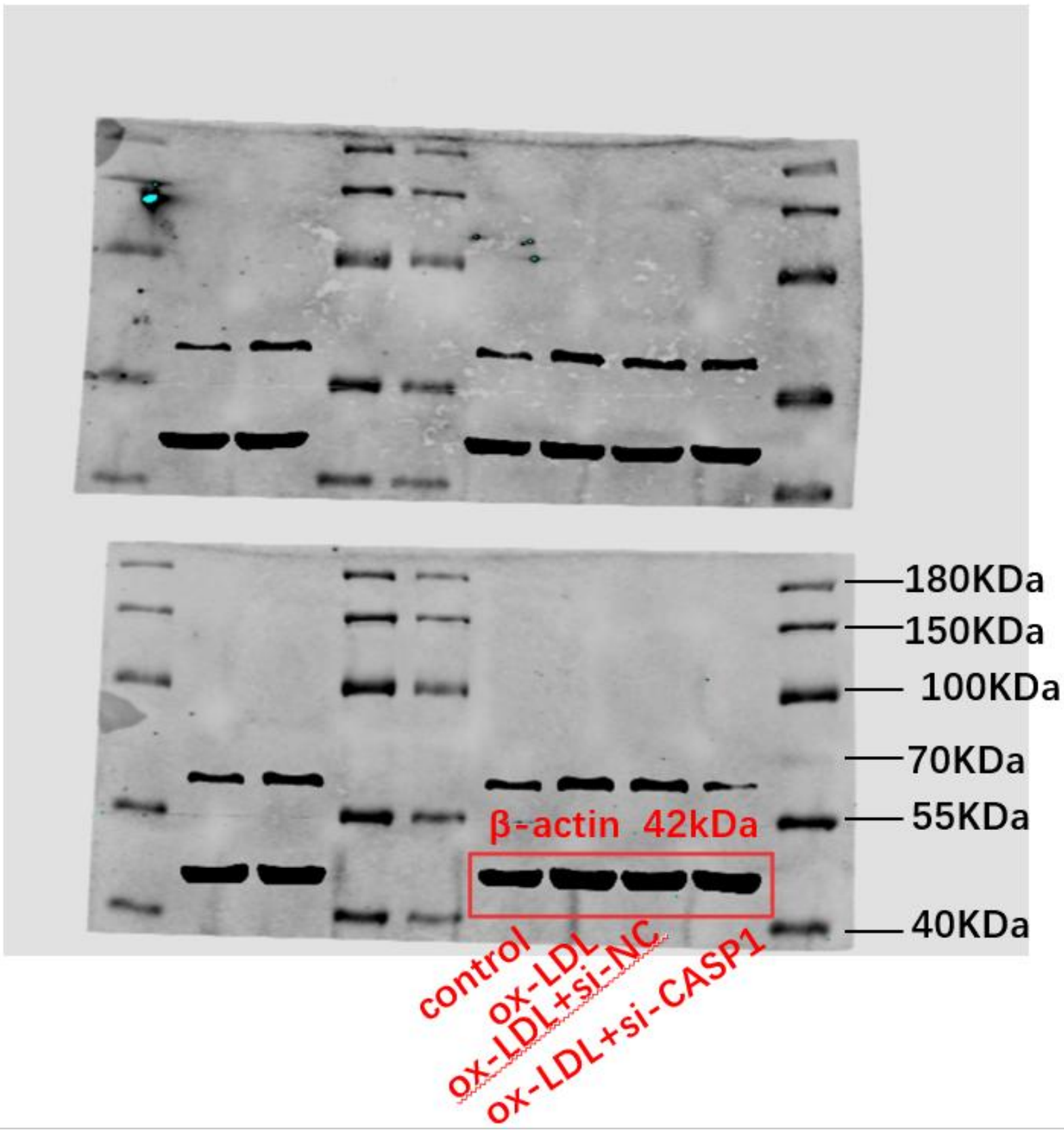

3-LC3II/LC3I

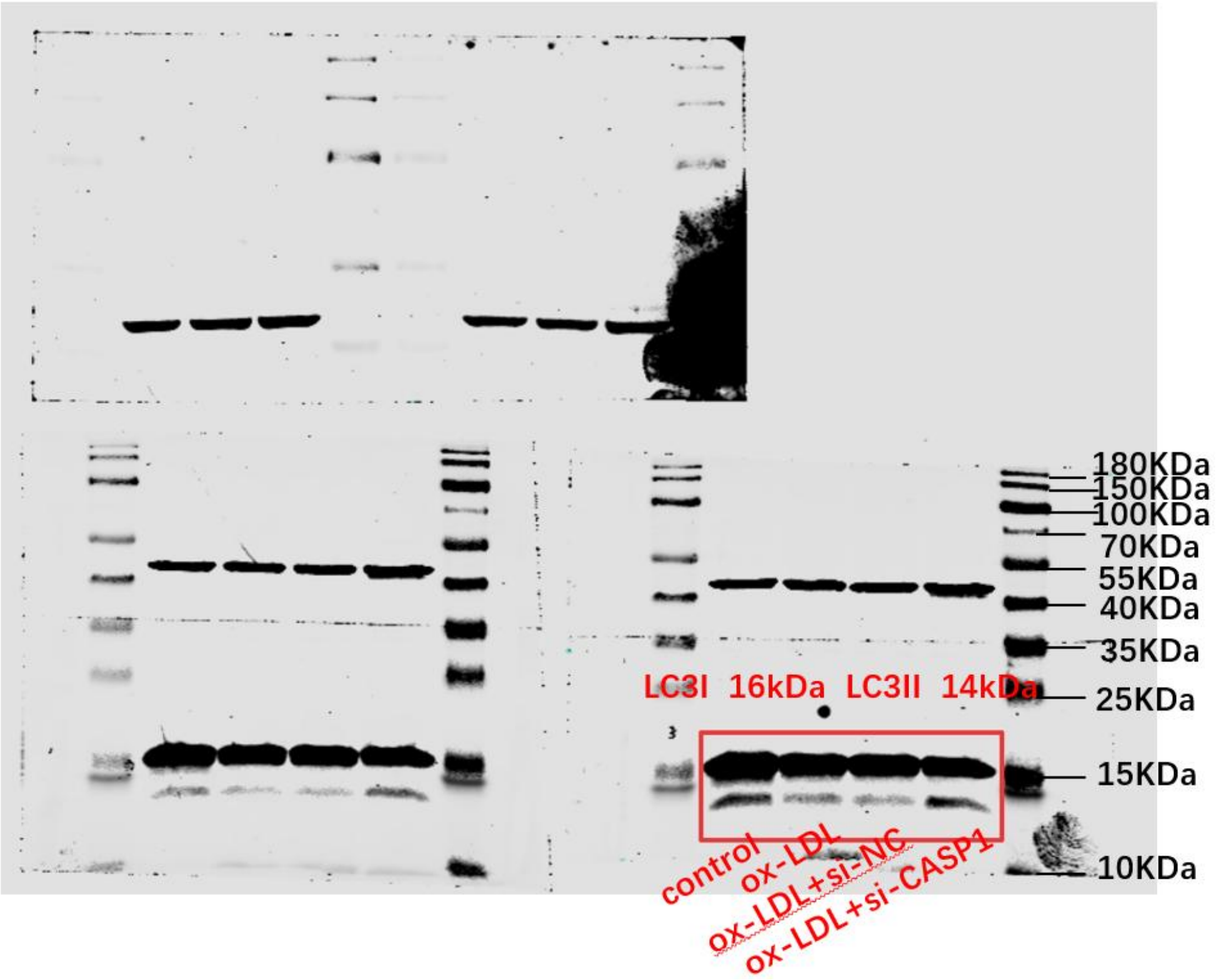

3-LC3II/LC3I-β-actin

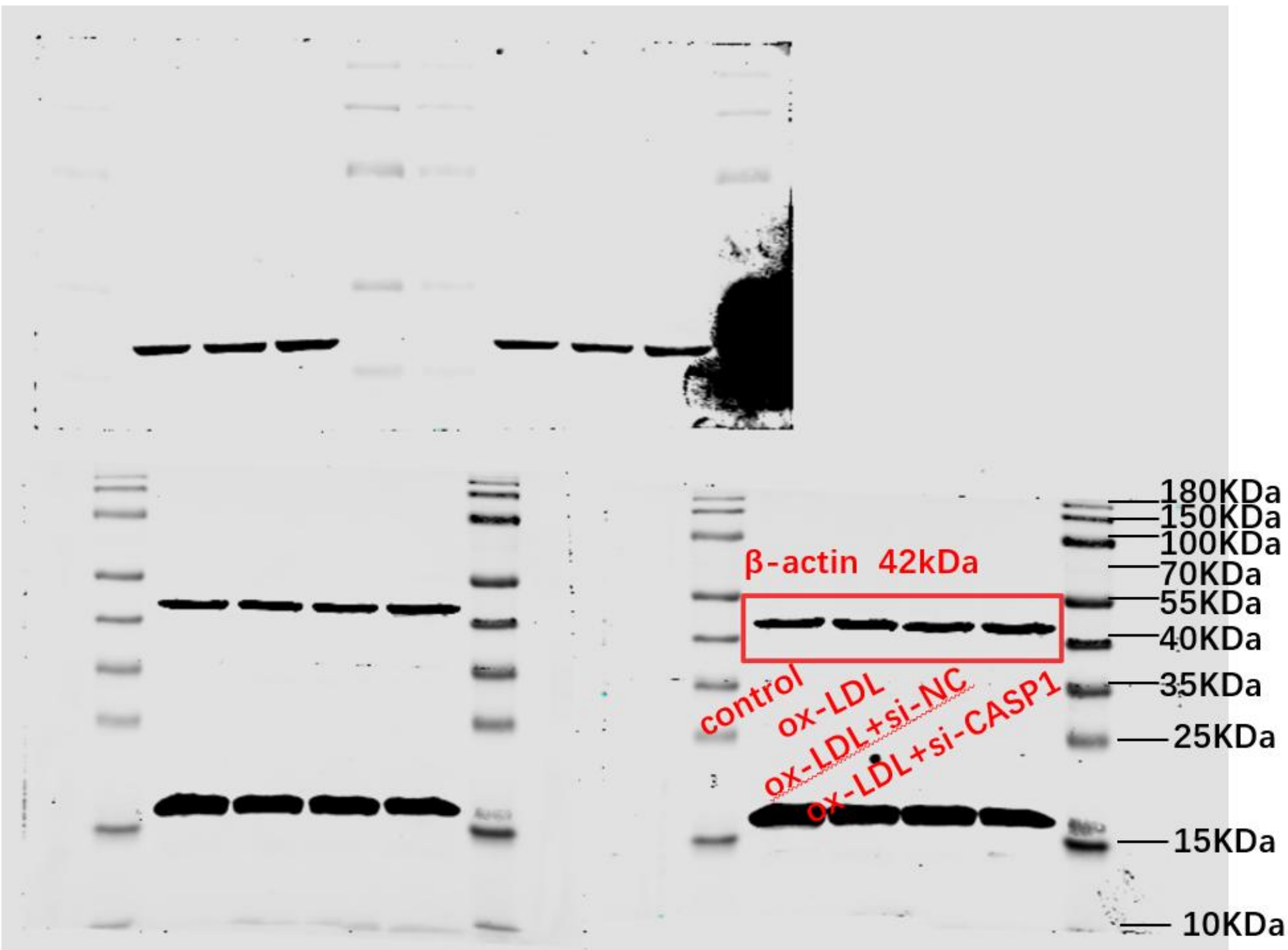

Figure 16

1-CASP1

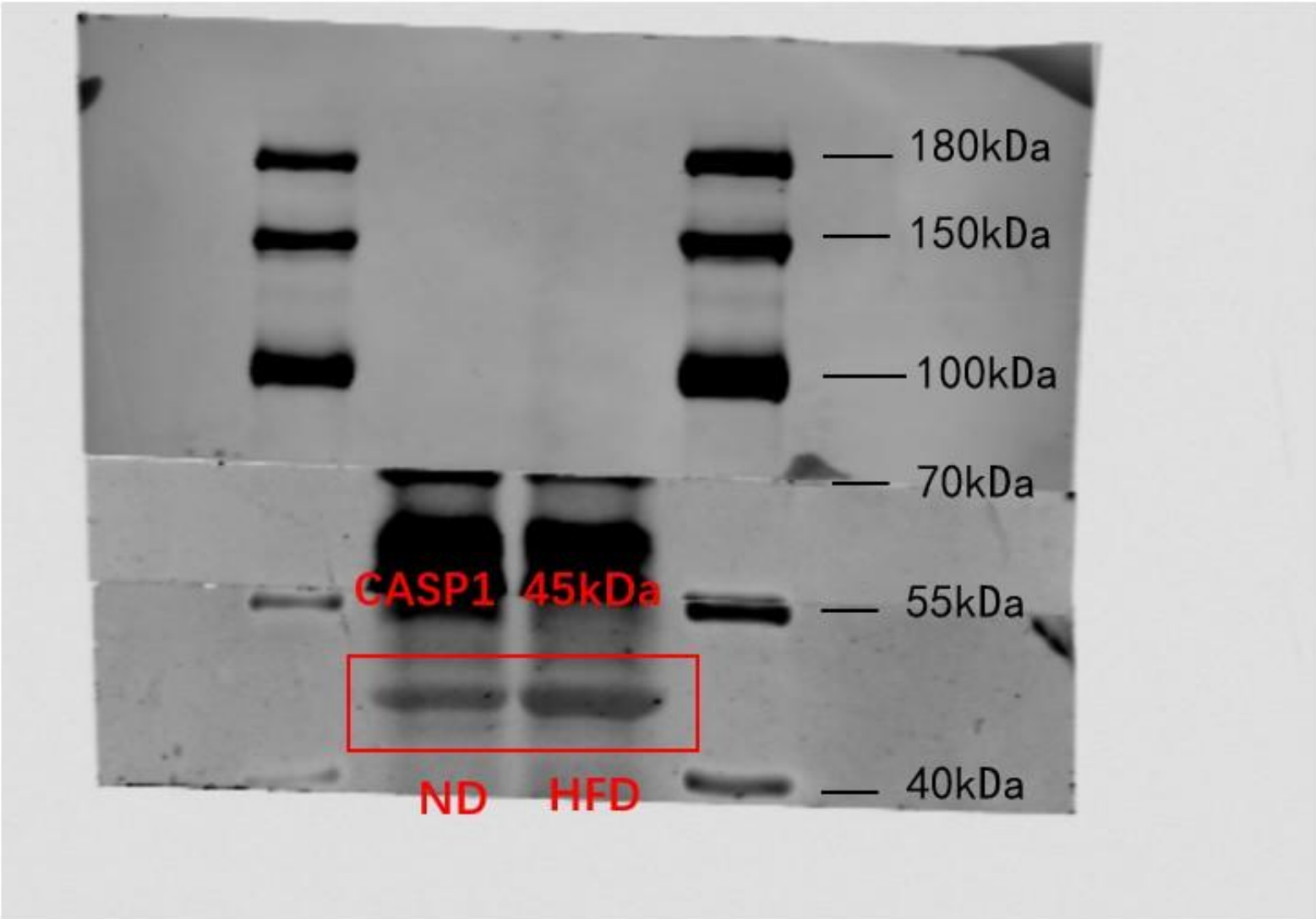

1-CASP1-actin

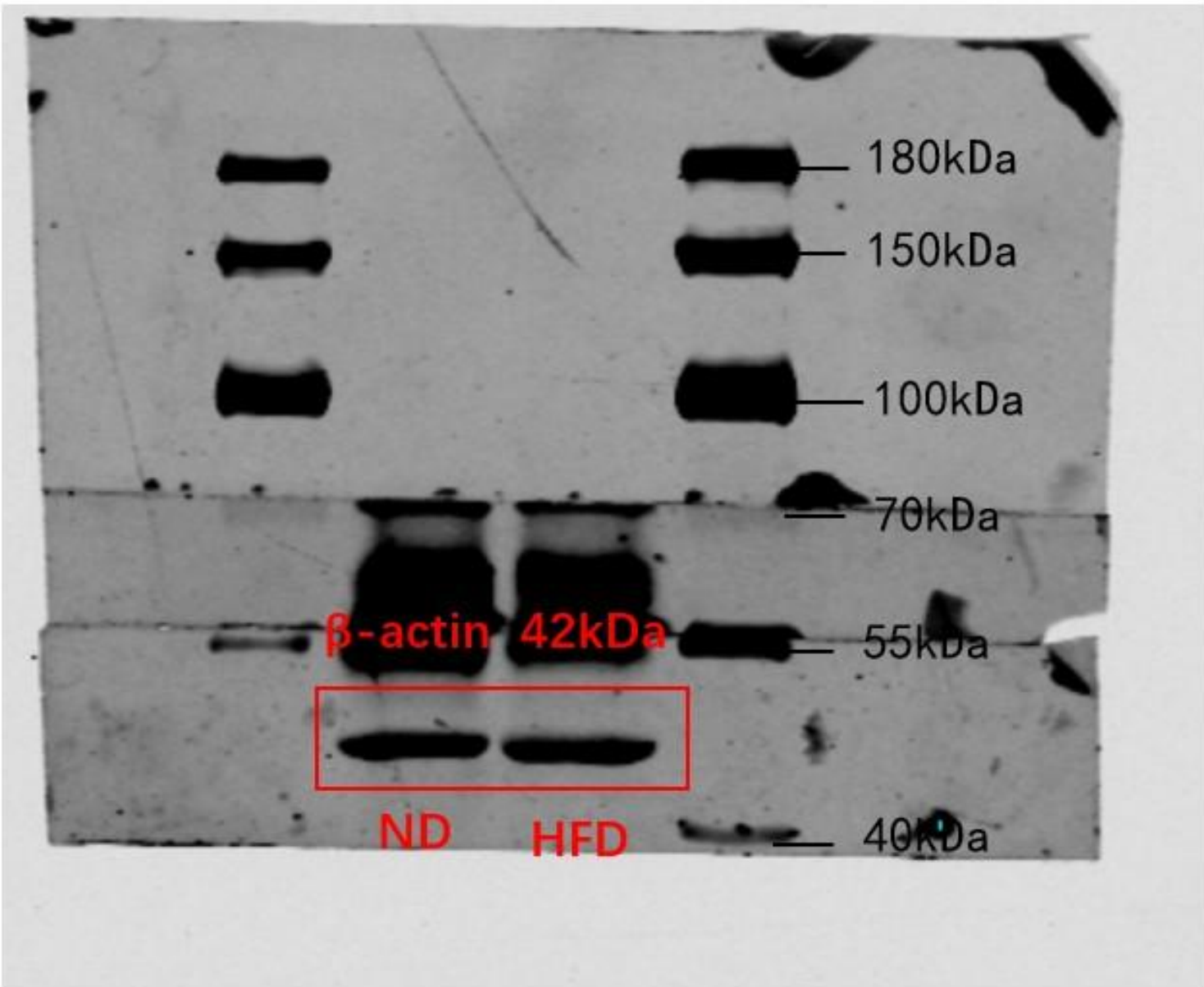

Supplement: Supplementary file 1 [file pone.0353352.s001.pdf]
